# Synthesis and Structure-Activity Relationship of Daphnetin Derivatives as Potent Antioxidant Agents

**DOI:** 10.3390/molecules23102476

**Published:** 2018-09-27

**Authors:** Yangliu Xia, Chen Chen, Yong Liu, Guangbo Ge, Tongyi Dou, Ping Wang

**Affiliations:** 1 School of Life Science and Medicine, Dalian University of Technology, Panjin 124221, China; xiayl@dlut.edu.cn (Y.X.); yliu@dlut.edu.cn (Y.L.); 2 College of Life Science, Dalian Minzu University, Dalian 116600, China; chenchen@dlnu.edu.cn; 3 Institute of Interdisciplinary Medicine, Shanghai University of Traditional Chinese Medicine, Shanghai 201203, China; gegbdicp@sohu.com

**Keywords:** daphnetin, antioxidant activity, radical scavenging effect, structure-activity relationship

## Abstract

In this study, daphnetin 1 was chosen as the lead compound, and C-3 or C-4-substituted daphnetins were designed and synthesized to explore the potential relationship between the antioxidant activities and the chemical structures of daphnetin derivatives. The antioxidant activities of the generated compounds were evaluated utilizing the free radical scavenging effect on 2,2′-diphenyl-1-picrylhydrazyl, 2,2′-azinobis-(3-ethylbenzthiazoline-6-sulfonate) cation, and the ferric reducing power assays, and were then compared with those of the standard antioxidant Trolox. The results showed that the catechol group was the key pharmacophore for the antioxidant activity of the daphnetins. The introduction of an electron-withdrawing hydrophilic group at the C-4 position of daphnetin enhanced the antioxidative capacity, but this trend was not observed for C-3 substitution. In addition, introduction of a a hydrophobic phenyl group exerted negative effects on the antioxidant activity in both the C-3 and C-4 substitutions. Among all of the derivatives tested, the most powerful antioxidant was 4-carboxymethyl daphnetin (compound **9**), for which the strongest antioxidant activity was observed in all of the assays. In addition, compound **9** also displayed strong pharmaceutical properties in the form of metabolic stability. To summarize, compound **9** holds great potential to be developed as an antioxidant agent with excellent antioxidant activity and proper pharmacokinetic behavior.

## 1. Introduction

Aerobic organisms form reactive oxygen species (ROS) as an unavoidable consequence of cell metabolism. In a healthy individual, there is an equilibrium between the natural antioxidative defense system and ROS. When the equilibrium is disrupted, ROS can induce both cellular damage responsible for aging and various pathologies in humans, such as rheumatoid arthritis, diabetes, and cancer [1,2,3]. A variety of powerful natural antioxidants have been identified that act as chemopreventive agents, which could prevent cancer through the action of antioxidants [4]. These compounds could also exert anti-mutagenicity, anti-carcinogenicity, and anti-aging properties, which are thought to originate from their antioxidant activities [5].

Natural compounds isolated from dietary or medicinal plants, which serve as rich sources of antioxidant agents, have been of increasing interest in recent decades. With the goal of searching for antioxidant components, the structure-activity relationships of some natural compounds have been extensively studied, generally focusing on the flavonoids and phenolic acids [6,7,8]. These reports demonstrated that the free radical scavenging and antioxidant activity of these compounds primarily depends on the type and position of the substituents on the aromatic ring of the molecules.

**Figure 1:**
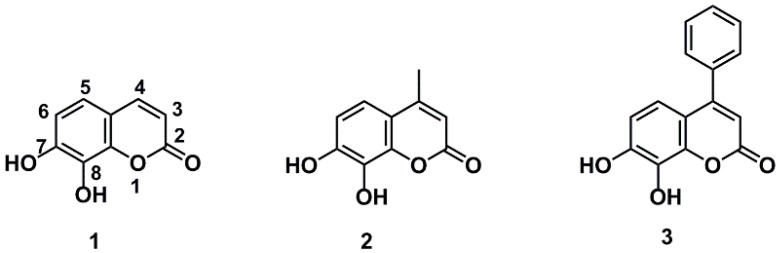
The structure of daphnetin and its analogues.

Coumarins are widely distributed in food and Chinese medicinal herbs, which are considered to be good natural sources of antioxidants. With excellent safety, high solubility, and good permeability, this family has received widespread attention [9,10,11,12,13]. As a representative coumarin, daphnetin (7,8-dihydroxycoumarin) has been approved as an adjunctive therapy for cardiovascular diseases in China since the 1980s, as well as receiving recognition as the marker compound for the quality control of the Zushima tablet, which has been used as a traditional Chinese medicine preparation to treat rheumatoid arthritis [14]. Daphnetin **1** and 4-methyl daphnetin **2** exhibited stronger activities in protecting mononuclear cells of umbilical blood from oxidative attack than resveratrol [15], and 4-methyl daphnetin **2** was found more effective in protection of human single cell DNA from oxidative attack than curcumin and resveratrol [16]. Although daphnetin **1** and its analogues (Figure 1) have been recognized to be good antioxidants, limited information is available on the structure–antioxidant activity relationship of daphnetin derivatives, which may provide vital information for the development of antioxidative agents based on daphnetin for the treatment or adjuvant treatment of oxidative stress-related diseases.

Therefore, daphnetin **1** was utilized in this study as a lead compound, and different groups with varied properties were introduced to the 3- or 4-position of daphnetin. The antioxidant activities of the derivatives were evaluated by examining their free radical scavenging effect using the FRAP, DPPH, and ABTS^+^ assays, and the potential structure-antioxidant activity relationships of daphnetins were discussed. In addition, the pharmaceutical activities of daphnetin derivatives, especially metabolic stability, which is the largest obstacle for the development of catechol coumarins as drugs, were investigated. In addition, we proposed a strategy of the simultaneous optimization of metabolic stability and pharmacological activity for these types of compounds. These findings could provide useful guidance to design and optimize potent antioxidants and the structural modifications of coumarin derivatives.

## 2. Results and Discussion

### 2.1. Chemistry

**Scheme 1:**
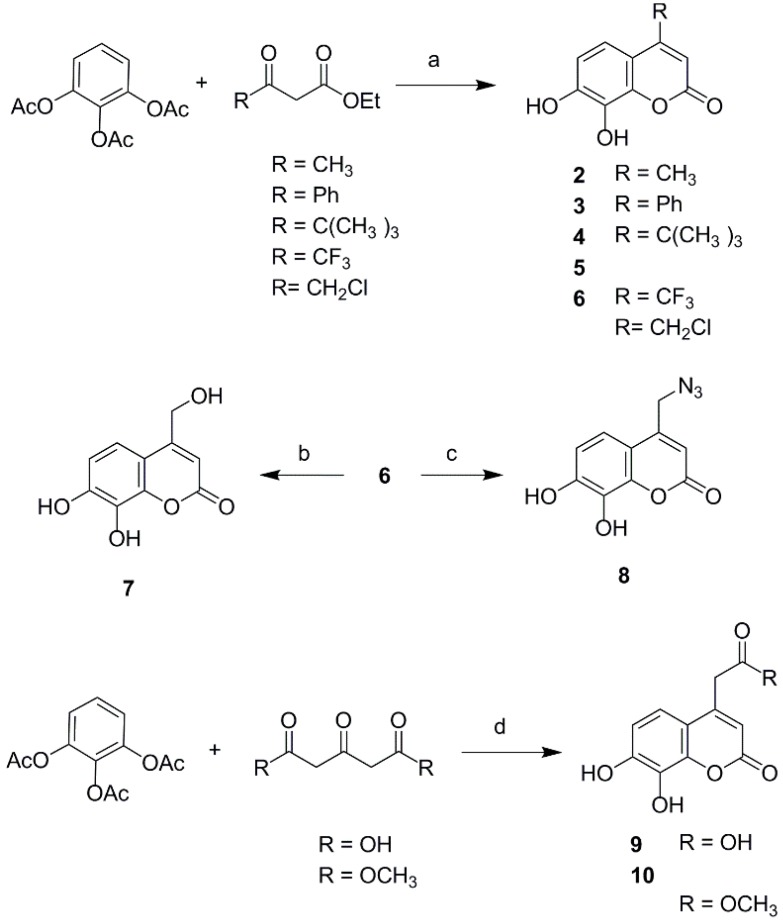
Synthesis of 4-substituted daphnetin derivatives. Reagents and conditions: (a) HClO_4_, 25–60 °C, 6–8 h; (b) DMF/H_2_O (1:3), reflux, 20 h; (c) DMF, NaN_3_, rt, 10 h; (d) HClO_4_, 50 °C, 4 h.

To explore the effects of different property groups and their position on daphnetin on its antioxidant activities, a series of 3- or 4-substituted daphnetin derivatives were designed. The substituents were selected mainly based on the electronic and lipophilic considerations defined by Craig′s plot [17]. In the present study, trifluoromethyl, phenyl and carboxylic ester were selected for their electron-withdrawing and hydrophobic property; carboxymethyl and carboxyl were selected to represent electron-withdrawing and hydrophilic substituents, while groups like tert-butyl and methyl represented electron-donating and hydrophobic substituents; hydroxymethyl, azidomethyl, and chloromethyl were representative of electron-donating and hydrophilic substitution. The derivatives of daphnetin were prepared via Knoevenagel condensation and acid catalyzed Pechmann reaction. First, the introduction of different property groups at the C-4 position of daphnetin is shown in Scheme 1. The compounds **2**–**6** were synthesized via acid catalytic Pechmann condensation. 1,2,3-Phenenyl triacetate was reacted with the correctly substituted *β*-ketoester under perchloric acid catalysis to produce compounds **2**–**6**. Subsequently, compound **7** and compound **8** were obtained from chloride **6** by refluxing in a mixture of dimethylformamide (DMF) and water (3:10, v:v) and treating with sodium azide, respectively. 4-Carboxylic daphnetin **9** and its ester **10** were obtained by treating 1,2,3-phenenyl triacetate with 1,3-acetonedicarboxylic acid and dimethyl 1,3-acetonedicarboxylate, respectively, in the present of perchloric acid.

**Scheme 2:**
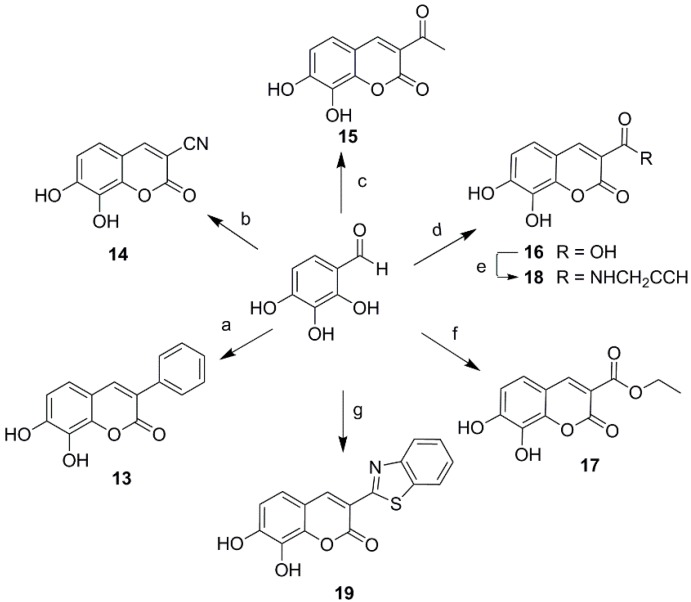
Synthesis of 3-substituted daphnetin derivatives. Reagents and conditions: (a) phenylacetic acid, AcONa, Ac_2_O, 6 h, 160 °C, 58%; (b) malononitrile, CH_3_COONH_4_, H_2_O, rt, 3 h; HCl (2 mol/L), 75 °C, 1 h, 80%; (c) ethyl acetoacetate, piperidine, MeOH, reflux, 3 h, 85%; (d) Meldrum′s acid, CH_3_COONH_4_, H_2_O, rt, 4 h, 83%; (e) 2-propynylamine, 1-hydroxybenzotriazole, EDCI, DMF, 5 °C, 5 h, 74%; (f) diethyl malonate, CH_3_COONH_4_, H_2_O, 50 °C, 5 h, 76%; (g) ethyl 2-(benzo[*d*]thiazol-2-yl) acetate, piperidine, MeOH, 40 °C, 5 h, 88%.

**Scheme 3:**
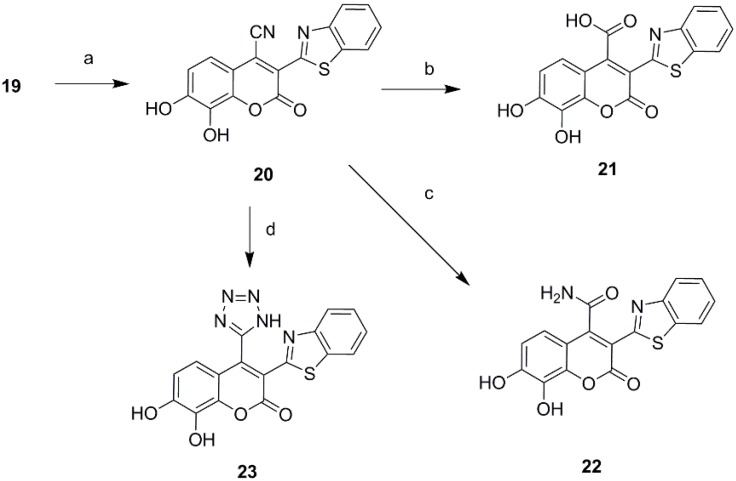
Synthesis of 3-benzothiazole daphnetin derivatives. Reagents and conditions: (a) NaCN, DMF, I_2_, 6 h, 58%; (b) H_2_SO_4_, H_2_O, 80 °C, 3 h, 36%. (c) H_2_SO_4_, H_2_O, 100 °C, 8 h; 60%; (d) ZnBr, NaN_3_, dioxane, 90 °C, 12 h, 42%.

Ultimately a series of different property groups, including phenyl, cyano, carbonyl, carboxyl, carboxylic ester and benzothiazole, were introduced to the C-3 site of daphnetin. All these compounds were prepared from 2,3,4-trihydroxybenzaldehyde through Knoevenagel condensation (Scheme 2) [18]. The detailed experimental procedures are described in the Supporting Information. In addition, a cyano group was introduced in the position of daphnetin **19** by an addition/elimination reaction similar to that described in reference [19] with a brief modification. Subsequently, a cyano group of **20** was hydrolyzed to acid **21** and amide **22**, as well as being converted to tetrazol **23** via the zinc-catalyzed [3 + 2] cycloadditon reaction (Scheme 3). Compounds **21**–**23** were purified using preparative high-performance liquid chromatography.

### 2.2. Antioxidant Activity Assay In Vitro

The antioxidant activity is influenced by many factors, which cannot be fully described with a single antioxidant assay. Thus, in this study, antioxidant activities in vitro were evaluated using the free radical scavenging effect on 2,2′-diphenyl-1-picrylhydrazyl (DPPH), 2,2′-azinobis-(3-ethylbenzthiazoline-6-sulfonate) cation (ABTS^+^). and the ferric reducing power (FRAP) assay, respectively, according to the literature with a slight modification [20,21,22,23,24]. The Trolox (6-hydroxy-2,5,7,8-tetramethylchroman-2-carboxylic acid; Sigma Aldrich, (St. Louis, MO, USA) standard solution (concentration range: 0–0.4 mmol/L) was analyzed under the same conditions as those used for the calibration. First, the reducing power of these compounds was evaluated using the FRAP assay by measuring the ability of an antioxidant to reduce ferric (III) ions to ferrous (II) ions. The results of the total equivalent antioxidant capacity (TEAC) were expressed as the Trolox equivalent, i.e., mmol equivalents of Trolox per mmol of sample (mmol Trolox/mmol sample).

**Figure 2:**
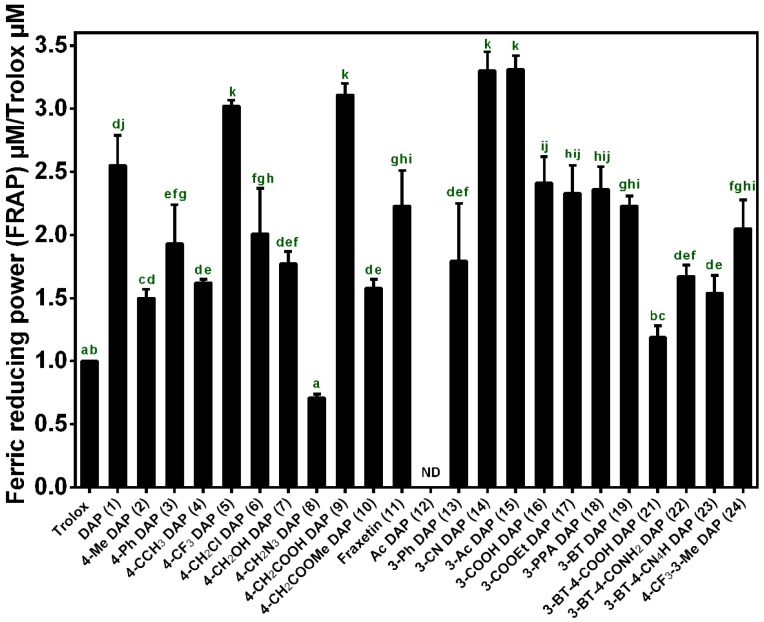
Ferric reducing power of daphnetin and its derivatives. Values marked by the same alphabet are not significantly different (*p* < 0.05).

As shown in Figure 2, all the daphnetin derivatives tested exhibited better reducing power than the standard antioxidant Trolox except compound **8** and **12** (TEAC_DPPH_ values >1). Daphnetin (DAP, **1**) exhibited a 2.5-fold higher reducing antioxidant power than that of the standard antioxidant Trolox. A three-fold increase in reducing antioxidant power was observed when trifluoromethyl **5** or carboxyl group **9** was introduced to the C-4 position of daphnetin, while other C-4 substituents, including phenyl **3**, methyl **2**, tert-butyl **4**, chloromethyl **6**, hydroxymethyl **7** and azidomethyl **8**, led to decreased activities of daphnetin but were still superior to Trolox except for the substitution of the azidomethyl group. The markedly decreased antioxidant activity of compound **8** is probably due to the ability of the azide group to cause the consumption of reducing agents and ultimately led to a lower reducing antioxidant power in comparison to the other substituents. In addition, the introduction of cyano **14** or acetyl group **15** improves the antioxidant activity of daphnetin, resulting in 3-fold higher antioxidant activity than that of Trolox. The C-3 carboxy substituent **16** of daphnetin displayed little effect on its antioxidant activity, and this situation did not change when the carboxyl group was esterified **17** or amidated **18**, which is quite different from the significant decrease in antioxidant activity observed when converting the C-4 carboxy to its ester **10**. In addition, after the introduction of a strong electron-withdrawing benzothiazole to the C-3 position, benzothiazole **19** exhibited a slightly reduced antioxidant activity. A simultaneous substitution at the C-4 position by carboxy group **21** led to a significant decrease in antioxidant activity, while this decline in activity could be alleviated by conversion of the carboxy group to amide **22** or its isostere tetrazolium **23**. However, it still had lower antioxidant activity compared with that of compound **19**. It is notable that the blocking of two hydroxyls by an acetyl group led to a complete loss of total reducing power, indicating that catechol moieties are key groups for the antioxidant activity of the daphnetin derivatives.

**Figure 3:**
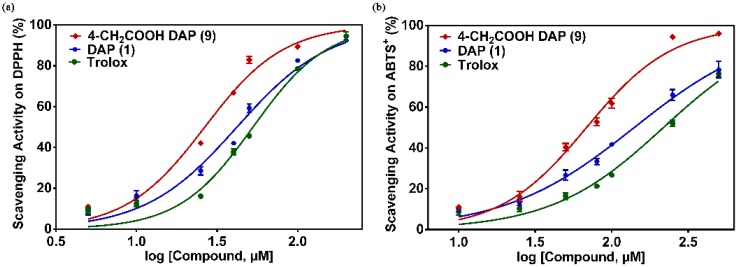
Concentration-response curve for the radical-scavenging activity of 4-CH_2_COOH DAP (**9**), daphnetin (DAP) (**1**), and positive control compound Trolox in 2,2′-diphenyl-1-picrylhydrazyl (DPPH) (**a**) and 2,2′-azinobis-(3-ethylbenzthiazoline-6-sulfonate) cation (ABTS^+^) (**b**) assays.

To evaluate the antioxidant activity of daphnetin derivatives in detail, the EC_50_ values of these compounds were determined using the DPPH and ABTS^+^ assays as shown in Table 1. In the DPPH assay, the EC_50_ value of DAP **1** is 46.20 μM, which is lower than that of Trolox (EC_50_ = 53.16 μM). Among these daphnetin derivatives, compound 4-carboxymethyl DAP **9** showed the strongest DPPH radical scavenging activity with EC_50_ values of 31.38 μM (Figure 3). Similar to the results of the FRAP assay, a significant increase in the DPPH radical scavenging activities of the daphnetins could be obtained when the C-4 position was substituted by strong electron-withdrawing groups, such as an acetate group, while few changes in the EC_50_ value were observed when the trifluoromethyl group was introduced to the C-4 position of daphnetin. Positive effects of the carboxyl group to the antioxidant activity of daphnetin also appeared when the substitution site was located in the C-3 position, which could be weakened by esterification or amidation of the carboxyl group with EC_50_ values of 37.67 μM, 43.98 μM, and 55.65 μM for compound **16**, **17**, and **18**, respectively. Interestingly, negative effects of cyano and acetyl on the DPPH radical scavenging activity of daphnetins appeared when the substitution site was switched from the C-4 to C-3 position, with the exception of an ethyl carboxylate group, in which a weakly enhanced activity was observed. In contrast to the same increased trend on the antioxidant activity of daphnetins when substituted by hydrophilic carboxyl, regardless of the C-3 or C-4 substitution, the substitution of hydrophobic phenyl exerted opposite effects towards the antioxidant activity of daphnetins depending on the substitution site. The antioxidant activity of 4-phenyl DAP **3** was lower than that of DAP **1**, while 3-phenyl DAP **13** was more effective. In addition, the DPPH radical scavenging activity of daphnetin dramatically decreased towards the blocked catechol groups, illustrating the important role of the catechol moieties in the depletion of DPPH.

In the ABTS^+^ radical assay, Table 1 and Figure 3 indicated that 4-carboxymethyl DAP **9** also showed strong scavenging activity on the ABTS^+^ radical, and their EC_50_ values were 72.38 μM. Similar to the FRAP assay and DPPH assay results, the ABTS^+^ radical scavenging ability of the daphnetins could be significantly enhanced by the substitution of a strong electron-withdrawing carboxyl group on the C-4 position. Taken together, 4-carboxymethyl DAP **9** is the most powerful antioxidant in these compounds and led to the best antioxidant activity observed in all the assays used in this study. In addition, diacetyl DAP **12** displayed very low antioxidative capacity with an EC_50_ value of 4562.49 μM. This provides additional evidence for the importance of the presence of two hydroxylated groups at the ortho position for the antioxidant activity of these compounds.

Based on these observations, a brief structure-activity relationship could be summarized. The existence of the phenolic groups and their positions are very important for the antioxidant activity of daphnetin. In particular, the catechol moiety plays a key role in enhancing the radical scavenging and ferric reducing activities. The introduction of an electron withdrawing hydrophilic group, such as a carboxyl group at the C-4 position of daphnetin, could enhance the antioxidative capacity significantly, but this trend was not observed in the C-3 substitution. However, the introduction of a hydrophobic phenyl group exhibited negative effects on the antioxidant activity in both the C-3 and C-4 substitutions. It is worth repeating that the carboxyl group displays more positive effects than all the other substituents, implying that the carboxyl group could serve as a pharmacophore for the antioxidant activity of daphnetins, and experiments to examine the antioxidant mechanism of carboxymethyl-substituted daphnetins are merited.

Although catechol coumarins have been reported to possess a variety of therapeutic benefits, this family suffers from low oral bioavailability due to poor metabolic stability [25,26]. The simultaneous improvement of utility as a drug and pharmacological activity is still a challenge for the structural optimization of lead compounds. From the view of the chemical structure, the catechol group is not only the pharmacophore of the coumarins but also the susceptible group for the attack of phase II metabolic enzymes. As phenolic hydroxyls, catechol coumarins primarily undergo conjugative reactions mediated by phase II metabolizing enzymes, including uridine-5-diphosphate glucuronosyl transferase (UGT) and catechol-O-methyltransferase (COMT) [27,28,29]. Considering that compound **9** exhibited the best antioxidant result in this study, the metabolic elimination t_1/2_ of compound **9** in the UGT and COMT incubation systems was investigated in more detail and compared with that of Compound **1** in vitro (Supporting Information). The results demonstrated a five-fold longer t_1/2_ for compound **9** than that of compound **1**, with t_1/2_ values of 96.5 min and 20.3 min, respectively (Figure S1). These results indicated that C-4 carboxyethyl substitution improves the metabolic stability of daphnetin. In summary, 4-carbethoxyl DAP **9** holds strong potential to be developed as an antioxidant agent with excellent antioxidant activity and good pharmacokinetic properties.

## 3. Materials and Methods

### 3.1. Materials and Measurements

The ^1^H NMR and ^13^C NMR spectra were recorded on a Bruker ARX (Bruker, Rheinstetten, Germany) 400 MHz or Bruker AMX 400 MHz or 600 MHz spectrometer in dimethyl sulfoxide (DMSO-*d*_6_) if not noted otherwise, and the chemical shifts were expressed as ppm using trimethylsilane (TMS) as an internal reference. High-resolution mass spectral (HRMS) analyses were measured on a TripleTOF™ Mass Spectrometer (TripleTOF 5600, SCIEX, Foster City, CA, USA). Daphnetin and its derivatives were analyzed using an ultra-fast liquid chromatography spectrometry system (Shimadzu, Kyoto, Japan) equipped with two LC-20AD pumps, a DGU-20A3 vacuum degasser, a SIL-20ACHT auto-sampler, a CTO-20AC column oven, and an SPD-M 20A diode-array detector (DAD). All the reagents used in the synthesis were obtained commercially and used without further purification. The reactions were monitored using thin layer chromatography (TLC) on glass packed precoated silica gel GF_254_ plates. Flash column chromatography was performed using silica gel (200–300 mesh), which was purchased from the Qingdao Ocean Chemical Co., Ltd (Qingdao, China). Daphnetin and 11 were purchased from Chengdu Pufei De Biotech Co., Ltd. (Sichuan, China).

### 3.2. Synthesis of 4-Substituted Daphnetin Derivatives

*General procedure for the synthesis of 4-substituted daphnetin derivatives* (**2**–**6**). Perchloric acid (5.0 mL) was added drop-wise at room temperature to a mixture of 1,2,3-phenenyl triacetate (5.0 mmol) and the correctly substituted *β*-ketoester (10.0 mmol) and stirred for 6–8 h. After TLC analysis indicated that the reaction was complete, the reaction mixture was slowly poured into a mixture of ice water (100 mL) with stirring. The resultant suspension was filtered, and the collected solid was washed with water and dried; the crude compound was recrystallized from methanol to produce the desired compound [30].

*7,8-Dihydroxy-4-methyl-2H-chromen-2-one* (**2**): Using the general procedure, 1,2,3-phenenyl triacetate was combined with ethyl acetoacetate in the presence of perchloric acid and left to react. The crude compound was recrystallized from the methanol to produce compound **2** as a light white solid with a yield of 78%. ^1^H NMR (600 MHz, DMSO) δ 10.05 (s, 1H), 9.28 (s, 1H), 7.09 (d, *J* = 8.6 Hz, 1H), 6.82 (d, *J* = 8.6 Hz, 1H), 6.13 (d, *J* = 1.1 Hz, 1H), 2.36 (s, 3H); ^13^C NMR (151 MHz, DMSO) δ 160.67, 154.40, 149.87, 143.79, 132.62, 115.95, 113.23, 112.58, 110.65, 18.72; HRMS (ESI) for C_10_H_8_O_4_, Calcd. 192.0423, found 192.0355 [M − H]^−^.

*7,8-Dihydroxy-4-phenyl-2H-chromen-2-one* (**3**): Using the general procedure, 1,2,3-phenenyl triacetate was combined with ethyl benzoylacetate in the presence of perchloric acid and left to react. The crude compound was recrystallized from the methanol to produce compound **3** as a white solid with a yield of 82 %. ^1^H NMR (600 MHz, DMSO) δ 10.19 (s, 1H), 9.42 (s, 1H), 7.77–7.32 (m, 5H), 6.88–6.65 (m, 2H), 6.14 (s, 1H); ^13^C NMR (151 MHz, DMSO) δ 160.56, 156.42, 150.11, 144.37, 135.94, 133.10, 129.94, 129.20, 128.90, 117.78, 112.81, 111.97, 110.72; HRMS (ESI) for C_15_H_10_O_4_, Calcd. 254.0579, found 253.0515 [M − H]^−^.

*7,8-Dihydroxy-4-tert-butyl-2H-chromen-2-one* (**4**): Using the general procedure, 1,2,3-phenenyl triacetate was combined with ethyl 4,4-dimethyl-3-oxovalerate in the presence of perchloric acid and left to react. The crude compound was recrystallized from methanol and purified using flash column chromatography on silica gel to produce compound **4** as a white solid with a yield of 39%. ^1^H NMR (600 MHz, DMSO) δ 10.21 (s, 1H), 9.25 (s, 1H), 7.34 (d, *J* = 8.6 Hz, 1H), 6.91 (d, *J* = 8.7 Hz, 1H), 6.04 (s, 1H), 1.33 (s, 9H); ^13^C NMR (151 MHz, DMSO) δ 177.58, 175.25, 150.54, 147.23, 133.35, 116.88, 115.31, 114.13, 105.45, 36.69, 28.04. HRMS (ESI) for C_13_H_14_O_4_, Calcd. 234.0892, found 233.0826 [M − H]^−^.

*7,8-Dihydroxy-4-trifluoromethyl-2H-chromen-2-one* (**5**): Using the general procedure, 1,2,3-phenenyl triacetate was combined with ethyl 4,4,4-trifluoroacetoacetate in the presence of perchloric acid and left to react. The crude compound was recrystallized from methanol to produce compound **5** as a yellow solid with a yield of 58%. ^1^H NMR (600 MHz, DMSO) δ 10.53 (s, 1H), 9.61 (s, 1H), 7.07 (dd, *J* = 8.8, 1.8 Hz, 1H), 6.92 (d, *J* = 8.8 Hz, 1H), 6.74 (s, 1H); ^13^C NMR (151 MHz, DMSO) δ 159.29, 151.03, 144.54, 140.76, 140.55, 133.40, 115.73, 113.61, 112.27, 106.45; HRMS (ESI) for C_10_H_5_F_3_O_4_, Calcd. 236.0140, found 245.0072 [M − H]^−^.

*7,8-Dihydroxy-4-chloromethyl-2H-chromen-2-one* (**6**): Using the general procedure, 1,2,3-phenenyl triacetate was combined with ethyl 4-chloroacetoacetate in the presence of perchloric acid and left to react. The crude compound was recrystallized from methanol to produce compound **6** as a white solid with a yield of 69%. ^1^H NMR (600 MHz, DMSO) δ 10.19 (s, 1H), 9.40 (s, 1H), 7.18 (d, *J* = 8.7 Hz, 1H), 6.85 (d, *J* = 8.7 Hz, 1H), 6.42 (s, 1H), 4.94 (s, 2H); ^13^C NMR (151 MHz, DMSO) δ 160.55, 151.86, 150.23, 144.15, 132.92, 115.95, 112.79, 111.42, 110.57, 41.95; HRMS (ESI) for C_10_H_7_ClO_4_, Calcd. 226.0033, found 224.9967 [M − H]^−^.

*7,8-Dihydroxy-4-hydroxymethyl-2H-chromen-2-one* (**7**): Chloride 6 (0.5 g, 2.2 mmol) was dissolved in a mixture of DMF (3 mL) and H_2_O (10 mL) with stirring and refluxed for 20 h. After TLC indicated that the reaction was complete, the mixture was diluted with water. The resultant suspension was filtered, and the collected solid was washed with water and dried. The crude compound was recrystallized from methanol to yield hydroxymethyl **7** as a light brown solid with a yield of 80%. ^1^H NMR (600 MHz, DMSO) δ 10.03 (s, 1H), 9.30 (s, 1H), 7.01 (d, *J* = 8.7 Hz, 1H), 6.78 (d, *J* = 8.6 Hz, 1H), 6.24 (t, *J* = 1.4 Hz, 1H), 5.55 (t, *J* = 5.5 Hz, 1H), 4.69 (d, *J* = 4.2 Hz, 2H); ^13^C NMR (151 MHz, DMSO) δ 161.03, 157.70, 149.75, 143.82, 132.75, 114.75, 112.65, 110.73, 106.85, 59.56; HRMS (ESI) for C_10_H_8_O_5_, Calcd. 208.0372, found 207.0304 [M − H]^−^.

*7,8-Dihydroxy-4-azidemethyl-2H-chromen-2-one* (**8**): Chloride 6 (0.5 g, 2.2 mmol) was dissolved in DMF (10 mL) and sodium azide (0.37 g, 5.5 mmol) was added and stirred overnight. After LC-MS indicated that the reaction was complete, the mixture was diluted with water. The resultant suspension was filtered, and the collected solid was washed with water and dried. The crude compound was recrystallized from methanol to yield azidemethyl **8** as a grey solid with a yield of 85%. ^1^H NMR (600 MHz, DMSO) δ 10.18 (s, 1H), 9.41 (s, 1H), 7.06 (d, *J* = 8.7 Hz, 1H), 6.83 (d, *J* = 8.7 Hz, 1H), 6.27 (s, 1H), 4.77 (d, *J* = 0.8 Hz, 2H); ^13^C NMR (151 MHz, DMSO) δ 160.47, 151.05, 150.26, 144.01, 132.88, 115.51, 112.82, 110.74, 109.76, 50.19; HRMS (ESI) for C_10_H_7_N_3_O_4_, Calcd. 233.0437, found 234.0513 [M + H]^+^.

*2-(7,8-Dihydroxy-2-oxo-2H-chromen-4-yl)acetic acid* (**9**): Using the general procedure, 1,2,3-phenenyl triacetate was combined with 1,3-acetonedicarboxylic acid in the presence of perchloric acid and left to react. The crude compound was recrystallized from methanol to yield compound **9** as a white solid with a yield of 78%. ^1^H NMR (400 MHz, DMSO) δ 12.75 (s, 1H), 10.11 (s, 1H), 9.35 (s, 1H), 7.03 (d, *J* = 8.7 Hz, 1H), 6.81 (d, *J* = 8.7 Hz, 1H), 6.22 (s, 1H), 3.80 (s, 2H); ^13^C NMR (100 MHz, DMSO) δ 171.23, 160.63, 150.99, 149.93, 143.95, 132.74, 116.03, 112.71, 112.57, 112.43, 37.88.

*Methyl 2-(7,8-dihydroxy-2-oxo-2H-chromen-4-yl)acetic acid* (**10**): Using the general procedure, 1,2,3-phenenyl triacetate was combined with dimethyl 1,3-acetonedicarboxylate in the presence of perchloric acid and left to react. The crude compound was recrystallized from methanol to yield compound **10** as a white solid with a yield of 83%. ^1^H NMR (600 MHz, DMSO) δ 10.12 (s, 1H), 9.36 (s, 1H), 7.01 (d, *J* = 8.7 Hz, 1H), 6.81 (d, *J* = 8.7 Hz, 1H), 6.24 (s, 1H), 3.92 (s, 2H), 3.65 (s, 3H); ^13^C NMR (151 MHz, DMSO) δ 170.20, 160.52, 150.33, 150.06, 143.96, 132.78, 116.00, 112.77, 112.57, 112.40, 52.64, 37.27. HRMS (ESI) for C_16_H_10_O_6_, Calcd. 250.0477, found 249.0413 [M − H]^−^.

*2-oxo-2H-Chromene-7,8-diyl diacetate* (**12**): A solution of daphnetin 1 (0.5 g, 2.8 mmol), pyridine (2.0 mL) and acetic anhydride (1 mL) in dichloromethane (DCM) (30 mL) was stirred overnight. After TLC indicated that the reaction was complete, the reaction mixture was poured into water and acidified with 1 mol/L hydrochloric acid. The reaction mixture was extracted with DCM (50 mL × 3). The combined organic layer was washed with water and brine, dried over anhydrous sodium sulfate, and concentrated by evaporation. The resultant suspension was recrystallized from methanol to yield ester **12** as a grey solid with a yield of 92%. ^1^H NMR (400 MHz, CDCl_3_) δ 10.22 (s, 1H), 8.40 (s, 1H), 7.59 (d, *J* = 8.6 Hz, 1H), 7.42–7.10 (m, 1H), 2.43 (s, 3H), 2.36 (s, 3H); ^13^C NMR (101 MHz, CDCl_3_) δ 187.26, 167.34, 167.06, 158.66, 148.72, 148.01, 144.99, 130.57, 127.95, 121.23, 120.13, 116.85, 20.69, 20.27.

### 3.3. Synthesis of 3-Substituted Daphnetin Derivatives

The synthetic routes of daphnetin derivatives **13**–**19** are illustrated in Scheme 2, and the details in the synthetic procedure of the compounds are described in the Supporting Information based on our previous study [18].

*3-(Benzo[d]thiazol-2-yl)-7,8-dihydroxy-2-oxo-2H-chromene-4-carboxylic acid* (**21**) and *3-(benzo[d]thiazol-2-yl)-7,8-dihydroxy-2-oxo-2H-chromene-4-carboxamide* (**22**): NaCN (0.24 g, 5.4 mmol) was added to a solution of 19 (0.5 g, 1.6 mmol) in DMF (10 mL) and stirred for 3 h at 50 °C. After the reaction was cooled to 0 °C, I_2_ (0.60 g, 2.4 mmol) was added and stirred for 5 h. After TLC indicated that the reaction was complete, the reaction mixture was poured into water and treated with an aqueous solution of NaHSO_3_. The reaction mixture was extracted with ethyl acetate. The combined organic layer was washed with water and brine, dried over anhydrous sodium sulfate, and concentrated by evaporation. The resultant suspension was recrystallized from methanol to yield compound **20** as a brown solid. Compound **20** was suspended in 60% sulfuric acid solution and heated to 100 °C for 8 h. The reaction mixture was extracted with abundant ethyl acetate and methanol. The combined organic layer was concentrated by evaporation and purified using preparative high-performance liquid chromatography to yield acid **21** with a yellowish-brown solid with a yield of 60%. ^1^H NMR (600 MHz, DMSO) δ 14.30 (s, 1H), 10.84 (s, 1H), 9.77 (s, 1H), 8.18 (d, *J* = 7.8 Hz, 1H), 7.97 (d, *J* = 8.1 Hz, 1H), 7.61–7.53 (m, 1H), 7.52–7.46 (m, 1H), 7.05 (d, *J* = 8.7 Hz, 1H), 7.00 (d, *J* = 8.7 Hz, 1H); ^13^C NMR (151 MHz, DMSO) δ 166.56, 160.36, 159.17, 152.45, 152.13, 143.79, 136.00, 132.90, 126.97, 125.92, 123.02, 122.48, 119.02, 114.52, 109.64; HRMS (ESI) for C_17_H_9_NO_6_S, Calcd. 355.0151, found 354.0084 [M − H]^−^.

Compound **20** was suspended in 60% sulfuric acid solution and heated to 80 °C for 3 h. The reaction mixture was extracted with abundant ethyl acetate and methanol. The combined organic layer was concentrated by evaporation and purified using preparative high-performance liquid chromatography to yield acid 22 with a yellowish-brown solid with a yield of 36%. ^1^H NMR (600 MHz, DMSO) δ 10.77 (s, 1H), 9.67 (s, 1H), 8.16 (d, *J* = 7.6 Hz, 1H), 8.06 (s, 1H), 8.00–7.90 (m, 2H), 7.60–7.53 (m, 1H), 7.52–7.45 (m, 1H), 7.14 (d, *J* = 8.8 Hz, 1H), 6.99 (d, *J* = 8.8 Hz, 1H); ^13^C NMR (151 MHz, DMSO) δ 166.50, 160.63, 159.42, 152.39, 152.09, 151.09, 143.76, 135.97, 132.67, 126.75, 125.81, 123.14, 122.31, 119.61, 114.28, 110.49, 109.76; HRMS (ESI) for C_17_H_9_N_2_O_5_S, Calcd. 354.0310, found 353.0245 [M − H]^−^.

*3-(Benzo[d]thiazol-2-yl)-7,8-dihydroxy-4-(1H-tetrazol-5-yl)-2H-chromen-2-one* (**23**): NaN_3_ (0.30 g 4.5 mmol) was added to a solution of 19 (0.25 g, 0.8 mmol) and ZnBr_2_ (0.45 g, 2.0 mmol) in dioxan (20 mL) and heated to 90 °C for 10 h. After TLC indicated that the reaction was complete, the reaction mixture was extracted with abundant ethyl acetate and methanol. The combined organic layer was concentrated by evaporation and purified using preparative high-performance liquid chromatography to yield acid **23** with a brown solid with a yield of 42%. ^1^H NMR (600 MHz, DMSO) δ 10.88 (s, 1H), 9.84 (s, 1H), 8.16–8.08 (m, 1H), 7.53 (d, *J* = 7.4 Hz, 1H), 7.48–7.36 (m, 2H), 6.90 (d, *J* = 8.8 Hz, 1H), 6.50 (d, *J* = 8.7 Hz, 1H); ^13^C NMR (151 MHz, DMSO) δ 159.54, 152.62, 151.88, 143.19, 135.88, 132.96, 126.89, 126.14, 123.07, 122.32, 119.23, 115.91, 114.29, 112.24, HRMS (ESI) for C_17_H_9_N_5_O_4_S, Calcd. 379.0375, found 378.0311 [M − H]^−^.

*7,8-Dihydroxy-3-methyl-4-(trifluoromethyl)-2H-chromen-2-one* (**24**): Drop-wise perchloric acid (5.0 mL) was added to a mixture of 1,2,3-phenenyl triacetate (0.5 g, 2.0 mmol) and ethyl 4,4,4-trifluoro-2-methyl-3-oxobutanoate (0.4 g, 0.2 mmol) at room temperature and stirred for 6 h. After TLC indicated that the reaction was complete, the reaction mixture was poured into water and extracted with ethyl acetate. The combined organic layer was washed with water and brine, dried over anhydrous sodium sulfate, and concentrated by evaporation. The residue was purified by column chromatography using silica gel to obtain compound **24** as a white solid with a yield of 28%. ^1^H NMR (600 MHz, DMSO) δ 10.28 (s, 1H), 9.48 (s, 1H), 7.14–6.97 (m, 1H), 6.87 (d, *J* = 9.0 Hz, 1H), 2.27 (q, *J* = 3.7 Hz, 3H); ^13^C NMR (151 MHz, DMSO) δ 161.34, 149.38, 142.52, 134.88, 134.68, 132.87, 124.76, 123.30, 122.91, 115.56, 113.29, 107.74, 14.05. HRMS (ESI) for C_11_H_7_F_4_O_4_, Calcd. 260.0296, found 259.0231 [M − H]^−^.

### 3.4. Antioxidant Activity Assay In Vitro

#### 3.4.1. DPPH Assay

Each sample in an ethanol solution (0.1 mL) was added to 3.9 mL DPPH ethanol solution (0.1 mmol/L). The solution was vortexed for 10 s and incubated at room temperature for 30 min. The absorbance of the resulting solution was measured at 517 nm (Hitachi U-2800 Spectrophotometer, Tokyo, Japan). The Trolox standard solution (concentration range: 0–0.4 mmol/L) in ethanol was analyzed under the same conditions as those used for calibration. The values of the DPPH radical scavenging activity were determined using a calibration curve of Trolox and expressed as the Trolox equivalent (TEAC_DPPH_, mmol Trolox/mmol sample).

#### 3.4.2. ABTS^+^ Assay

ABTS^+^ solution was produced by reacting 7 mmol/L ABTS water solution (10 mL) with 2.45 mmol/L potassium persulphate (10 mL). The mixture was incubated in the dark at room temperature for 15–16 h before use. To evaluate the antioxidant activity, the ABTS^+^ solution was diluted with ethanol until its absorbance reached 0.7 at 734 nm. Each sample solution (0.1 mL) was added to 3.9 mL ABTS^+^ solution, and the absorbance was measured at room temperature after 3 min. The Trolox standard solution (concentration range: 0–0.4 mmol/L) in ethanol was analyzed under the same conditions as those used for the calibration. Values of the ABTS radical scavenging activity were determined using a calibration curve of Trolox and expressed as the Trolox equivalent (TEAC_ABTS_, mmol Trolox/mmol sample).

#### 3.4.3. FRAP Assay

The FRAP (ferric reducing antioxidant power) reagent was prepared with acetate buffer (300 mmol/L, pH 3.6), 2,4,6-tripyridyl-s-triazine (TPTZ) (10 mmol/L in HCl, 40 mmol/L) and FeCl_3_ (20 mmol/L). The proportions were 10:1:1 (v:v:v), respectively. Each sample solution was added to the FRAP reagent (1:30, v:v) and incubated at 37 °C for 5 min. The absorbance was determined at 593 nm using a spectrophotometer (Hitachi U-2800 Spectrophotometer, Tokyo, Japan). The Trolox standard solution (concentration range: 0–0.4 mmol/L) in ethanol was analyzed under the same conditions as those used for the calibration. The values of the reducing power were determined using a calibration curve of Trolox and expressed as the Trolox equivalent (TEAC_FRAP_, mmolTrolox/mmol sample).

### 3.5. Statistical Analysis

All the experiments were conducted in triplicate, and the data were analyzed using SPSS software (Version 14.0; SPSS, Chicago, IL, USA). A one-way analysis of variance and least significant difference (LSD) were used to differentiate the mean values.

## 4. Conclusions

In summary, daphnetin was chosen as the lead compound and more than twenty derivatives were designed and synthesized by structural modification on C-3 and/or C-4 position of daphnetin. The antioxidant activities of these compounds were assayed. Meanwhile, the potential structure–inhibition relationships of these daphnetin derivatives were well-summarized and discussed. The results clearly demonstrated that the catechol group of daphnetins was essential for antioxidant activity, while introduction of an electron withdrawing hydrophilic group at the C-4 position of daphnetin might be beneficial for antioxidant effect. Among all the tested compounds, 4-carboxymethyl daphnetin (compound **9**) exhibited the strongest antioxidant activity in all of the assays, implying that this compound could serve as an ideal lead compound for the development of a potent antioxidant. Further investigation on the metabolic stability demonstrated compound **9** exerted a three-fold longer t_1/2_ than that of compound 1 in the UGT and COMT incubation systems in vitro. All these findings are very useful for medicinal chemists to design and develop potent antioxidants based on the structural modifications of coumarin derivatives.

## Figures and Tables

**Table 1:** Antioxidant activity of daphnetin derivatives tested in 2,2′-diphenyl-1-picrylhydrazyl (DPPH) and 2,2′-azinobis-(3-ethylbenzthiazoline-6-sulfonate) cation (ABTS^+^) assays.DAP = daphnetin.

Compound	DPPH (EC_50_/μM)	ABTS^+^ (EC_50_/μM)
Trolox	53.16 ± 2.02 ^ab^	241.33 ± 2.52 ^k^
DAP (**1**)	46.20 ± 1.45 ^ab^	159.34 ± 4.63 ^fghi^
4-Me DAP (**2**)	85.63 ± 2.13 ^c^	224.50 ± 1.94 ^ijk^
4-Ph DAP (**3**)	55.60 ± 0.78 ^ab^	198.33 ± 28.18 ^ijk^
4-CCH_3_ DAP (**4**)	65.50 ± 2.84 ^bc^	234.68 ± 2.03 ^k^
4-CF_3_ DAP (**5**)	45.69 ± 1.26 ^ab^	75.23 ± 1.58 ^ab^
4-CH_2_Cl DAP (**6**)	46.57 ± 2.57 ^ab^	107.39 ± 6.72 ^abcde^
4-CH_2_OH DAP (**7**)	61.99 ± 1.43 ^bc^	184.94 ± 2.04 ^hij^
4-CH_2_N_3_ DAP (**8**)	62.17 ± 3.73 ^bc^	238.31 ± 4.47 ^k^
4-CH_2_COOH DAP (**9**)	31.38 ± 0.68 ^a^	72.31 ± 2.70 ^a^
4-CH_2_COOMe DAP (**10**)	50.20 ± 1.12 ^ab^	222.70 ± 1.84 ^jk^
Fraxetin (**11**)	45.59 ± 1.48 ^ab^	173.07 ± 4.83 ^ghi^
Ac DAP (**12**)	698.04 ± 5.22 ^f^	4562.49 ± 50.1 ^l^
3-Ph DAP (**13**)	43.67 ± 2.08 ^ab^	108.01 ± 1.42 ^abcde^
3-CN DAP (**14**)	47.02 ± 2.05 ^ab^	95.80 ± 4.18 ^abcd^
3-Ac DAP (**15**)	50.62 ± 0.61 ^ab^	80.52 ± 0.61 ^abc^
3-COOH DAP (**16**)	37.67 ± 1.16 ^ab^	145.67 ± 5.77 ^efgh^
3-COOEt DAP (**17**)	43.98 ± 0.89 ^ab^	138.88 ± 0.14 ^defg^
3-PPA DAP (**18**)	55.65 ± 1.48 ^ab^	91.25 ± 2.29 ^abcd^
3-BT DAP (**19**)	71.31 ± 2.40 ^bc^	86.57 ± 3.17 ^abcd^
3-BT-4-COOH DAP (**21**)	130.50 ± 3.54 ^d^	108.33 ± 6.35 ^abcde^
3-BT-4-CONH_2_ DAP (**22**)	66.82 ± 1.38 ^bc^	122.34 ± 4.41 ^bcdef^
3-BT-4-CN_4_H DAP (**23**)	167.85 ± 11.63 ^e^	121.72 ± 0.22 ^cdef^
4-CF_3_-3-Me DAP (**24**)	53.76 ± 2.25 ^ab^	107.21 ± 6.23 ^abcde^

Values marked by the same alphabet are not significantly different (*p* < 0.05).

## Supplementary Materials

The following are available online, Figure S1: Plot of incubation time vs. natural log percent remaining.
