# Supplementary material for: Synthesis and Structure-Activity Relationship of Daphnetin Derivatives as Potent Antioxidant Agents
Source: Molecules. 2018 Sep 27;23(10):2476. doi: 10.3390/molecules23102476 (PMC6222747; doi:10.3390/molecules23102476)

Supplementary material

for

Synthesis and Structure-Activity Relationship of Daphnetin Derivatives as Potent Antioxidant Agents

Yangliu Xia [1,+], Chen Chen [2,+], Yong Liu [1], Guangbo Ge [3], Tongyi Dou [1,*], Ping Wang [3,*]

[1] School of Life Science and Medicine, Dalian University of Technology, Panjin 124221, China

[2] College of Life Science, Dalian Minzu University, Dalian 116600, China

[3] Institute of Interdisciplinary Medicine, Shanghai University of Traditional Chinese Medicine, Shanghai 201203, China

[*]Corresponding author.

Ping Wang, Shanghai University of Traditional Chinese Medicine, No. 1200 Cailun Road, Shanghai 201210, China. Tel: +86-21-51323184; E-mail: s200541025@126.com

Tongyi Dou, Dalian University of Technology, No.2 Dagong Road, Panjin 124221, China. Tel.: +86-427-2631425; E-mail: douty@dicp.ac.cn

[+] These authors contributed equally to this work.

Table of Contents

S1. Experimental procedure for compound **13~19** ……………………………………………….S3-5

S2. Metabolic stability assay in vitro …………………………………………….…………… S6

Figure S1. Plot of incubation time vs. natural log percent remaining…………………………… S7

S3. ^{1}H NMR and ^{13}C NMR spectra for the synthetic compounds S8-27

*7,8-dihydroxy-3-phenyl-2H-chromen-2-one (**13**)* A mixture of 2,3,4-trihydroxybenzaldehyde (1.0 g, 6.5 mmol), phenylacetic acid (1.3 g, 9.7 mmol), and sodium acetate(1.6 g 19.5 mmol) in 20 mL acetic anhydride was heated with stirring at 160 °C for 6 h. After removal of acetic acid by distillation, the resulting mixture was treated with 2.0 N hydrochloric acid, and the precipitates were collected by filtration. The dried product was recrystallized from ethanol to afford compound **13** (950 mg) in 58% yield as a pale yellow solid. ^{1}H NMR (400 MHz, DMSO) δ 10.16 (s, 1H), 9.43 (s, 1H), 8.12 (s, 1H), 7.72 (d, *J* = 7.5 Hz, 2H), 7.41 (dt, *J* = 25.7, 7.2 Hz, 3H), 7.12 (d, *J* = 8.4 Hz, 1H), 6.86 (d, *J* = 8.4 Hz, 1H), ^{13}C NMR (101 MHz, DMSO) δ 160.52, 150.09, 143.67, 142.16, 135.67, 132.31, 128.78, 128.61, 128.42, 122.42, 119.68, 113.27, 113.26; ESI-MS: M = 254.0, found 255.0 $[M+H]^{+}$.

*7,8-dihydroxy-2-oxo-2H-chromene-3-carbonitrile (**14**)* 2,3,4-trihydroxybenzaldehyde (1.0 g, 6.5 mmol), malononitrile (0.64 g, 9.7 mmol) and ammonium acetate (0.75 g, 9.7 mmol) were mixed in 20 mL of water. The reaction mixture was stirred under the room temperature for 3h. The resulted solid was filtered and washed three times with 10 mL of water. Next, the products were added in 20 mL 3N hydrochloric acid solution, and the reaction mixture was stirred at 75 ^{o}C for 1h. After reaction, the reaction mixture was cooled to room temperature. The resulted solid was filtered, and recrystallized from methanol to afford compound **14** (1.05 g) in 80% yield as a red solid. ^{1}H NMR (400 MHz, DMSO) δ 10.27 (s, 2H), 8.75 (s, 1H), 7.18 (d, *J* = 8.5 Hz, 1H), 6.92 (d, *J* = 8.5 Hz, 1H); ^{13}C NMR (101 MHz, DMSO) δ 158.02, 154.33, 154.07, 144.63, 132.78, 121.92, 115.85, 114.20, 111.54, 96.24; ESI-MS: M = 203.0, found 202.0 $[M-H]^{-}$.

*3-acetyl-7,8-dihydroxy-2H-chromen-2-one (**15**)* To a solution of 2,3,4-trihydroxybenzaldehyde (1.0 g, 6.5 mmol) and ethyl acetoacetate (1.0 ml, 8.5 mmol) in MeOH (15 ml) was added piperidine (0.1 ml). Then the reaction mixture was heated to reflux for 5 h. After cooling, the reaction mixture was poured slowly into a mixture of ice-water (50 ml) with stirring. The resultant suspension was filtered, and the collected solid was washed with water and dried, then the crude compound was recrystallized from methanol to afford compound **15** (1.2 g) in 85% yield as a yellow solid. ^{1}H NMR

(400 MHz, DMSO) δ 10.71 (s, 1H), 9.60 (s, 1H), 8.55 (s, 1H), 7.31 (d, *J* = 8.5 Hz, 1H), 6.88 (d, *J* = 8.5 Hz, 1H), 2.57 (s, 3H); ^{13}C NMR (101 MHz, DMSO) δ 195.26, 159.50, 153.14, 148.95, 145.41, 132.27, 122.75, 119.46, 113.92, 112.02, 30.58; ESI-MS: M = 220.0, found 219.0 $[M-H]^-$.

*7,8-dihydroxy-2-oxo-2H-chromene-3-carboxylic acid (**16**)* 2,3,4-trihydroxybenzaldehyde (1.0 g, 6.5 mmol), Meldrum's acid (1.4 g, 9.7 mmol) and ammonium acetate (0.75 g, 9.7 mmol) were mixed in 20 mL of water. The reaction mixture was stirred under the room temperature for 4h. After reaction, 2.0 N hydrochloric acid was added to adjust pH to 2-3. The resulted solid was filtered and washed three times with water. Next, the product was recrystallized from methanol to afford compound **16** (1.45 g) in 83% yield as a yellow solid. ^{1}H NMR (400 MHz, DMSO) δ 8.42 (s, 1H), 7.17 (d, *J* = 8.6 Hz, 1H), 6.79 (d, *J* = 8.5 Hz, 1H); ^{13}C NMR (101 MHz, DMSO) δ 166.11, 159.87, 155.84, 147.92, 144.41, 132.74, 122.04, 114.65, 112.72, 111.29; ESI-MS: M = 222.0, found 221.0 $[M-H]^-$.

*Ethyl 7,8-dihydroxy-2-oxo-2H-chromene-3-carboxylate (**17**)* 2,3,4-trihydroxybenzaldehyde (1.0 g, 6.5 mmol), diethyl malonate (1.5 ml, 9.7 mmol) and ammonium acetate (0.75 g, 9.7 mmol) were mixed in 25 mL of water. The reaction mixture was heated to 50 ^{o}C for 4h. After cooling, 2.0 N hydrochloric acid was added to adjust pH to 2-3. The resultant suspension was filtered, and the collected solid was washed with water and dried, then the crude compound was recrystallized from methanol to afford compound **17** (1.2 g) in 76% yield as a pale yellow solid. ^{1}H NMR (400 MHz, DMSO) δ 10.65 (s, 1H), 9.57 (s, 1H), 8.64 (s, 1H), 7.28 (d, *J* = 8.5 Hz, 1H), 6.87 (d, *J* = 8.5 Hz, 1H), 4.27 (q, *J* = 7.1 Hz, 2H), 1.30 (t, *J* = 7.1 Hz, 3H); ^{13}C NMR (101 MHz, DMSO) δ 163.50, 156.86, 153.01, 150.49, 145.32, 132.24, 122.09, 113.74, 112.31, 111.63, 61.25, 14.61; ESI-MS: M = 250.0, found 249.1 $[M-H]^-$.

*3-(benzo[d]thiazol-2-yl)-7,8-dihydroxy-2H-chromen-2-one (**19**)* To a solution of 2,3,4-trihydroxybenzaldehyde (1.0 g, 6.5 mmol) and ethyl 2-(benzo[*d*]thiazol-2-yl) acetate (1.6 ml, 7.2 mmol) in MeOH (15 ml) was added piperidine (0.1 ml). Then the reaction mixture was heated to 40 °C and stirred for 5 h. After cooling, the reaction mixture was poured

slowly into a mixture of ice-water (50 ml) with stirring. The resultant suspension was filtered, and the collected solid was washed with water and dried, then the crude compound was recrystallized from methanol to afford **19** (1.8 g) in 88% yield as a brown solid. ^{1}H NMR (400 MHz, DMSO) δ: 10.59 (s, 1H), 9.68 (s, 1H), 9.11 (s, 1H), 8.15 (d, *J* = 8.0 Hz, 1H), 8.04 (d, *J* = 8.4 Hz, 1H), 7.55 (t, *J* = 8.0 Hz, 1H), 7.44 (t, *J* = 8.8 Hz, 1H), 7.43 (d, *J* = 8.8 Hz, 1H), 6.95 (d, *J* = 8.8 Hz, 1H); ^{13}C NMR (100 MHz, DMSO) δ: 112.7, 114.2, 114.7, 122.0, 122.5, 122.6, 125.4, 126.9, 132.6, 136.1, 143.7, 144.1, 152.5, 160.2, 161.0; HRMS (ESI) Calcd for $C_{16}H_{8}NO_{4}S^{-}$ ($[M-H]^{-}$) 310.0174, found 310.0178.

Metabolic stability assay in vitro

To determine the effects of a combination of glucuronidation and methylation, daphnetin and compound **9** were incubated with S9 fraction in the presence of two cofactors (2 mM UDPGA and 0.2 mM SAM). The incubation mixture contained pooled human S9 fraction (0.02 mg mL^{-1} protein), 5 mM $MgCl_2$, 2 mM UDPGA, 0.2 mM SAM and 5 mM daphnetin derivatives (dissolved in DMSO, final concentration 1%) in 200 mL of 50 mM Tris buffer (pH 7.4), as described in the reported manuscript. Controls were incubated in the absence of UDPGA and SAM. After incubation at 37 °C for 0 to 60 min, the reactions were terminated by adding 200 mL of cold acetonitrile. The samples were centrifuged at 20 000 g for 20 min. The supernatants (200 mL) were subjected to assay for the time-dependent esculetins depletion using HPLC.

Daphnetin and compound 9 were analyzed by a ultra-fast liquid chromatography spectrometry system (Shimadzu, Kyoto, Japan), equipped with two LC-20AD pumps, a DGU-20A3 vacuum degasser, a SIL-20ACHT auto-sampler, a CTO-20AC column oven, an SPD-M 20A diode-array detector (DAD), a CBM-20A communications bus module, a mass detector (2010 EV) with an electrospray ionization (ESI) interface, and a computer equipped with UFLC-MS Solution version 3.41 software. A Hedera C18 (150.0 mm × 2.1 mm, 3 μm) analytical column was used and column temperature was kept at 40 °C. The mobile phase was acetonitrile (A) and 0.2% formic acid water (B) at a flow rate of 0.4 mL min^{-1}, with a gradient: 0-6.0 min, 95% B-65% B; 6.0-9.0 min, 5% B; 9.0-15 min, balance to 95% B.

To determine the value of in vitro in vitro half-life ($t_{1/2}$), peak areas of parent drug at each time point are tabulated and expressed as a percent of the peak area at the zero minute time point, which represents 100%. $t_{1/2}$ was determined using the equation: $t_{1/2} = \ln 2/-k$, where k represents the terminal elimination rate constant and is calculated as the negative slope of the line defined by the linear regression of the natural log loss of substrate and incubation time (Fig. S1).

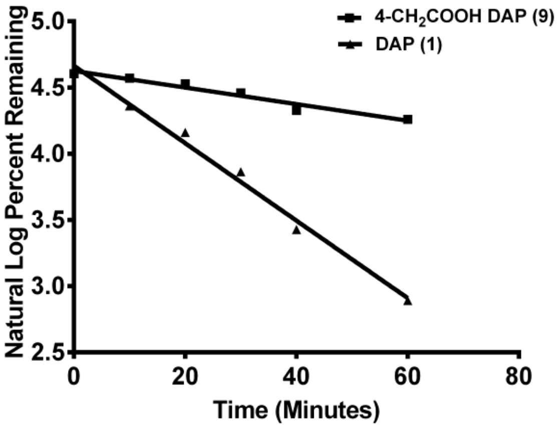

Figure S1. Plot of incubation time vs. natural log percent remaining.

S1. ^{1}H NMR and ^{13}C NMR spectra for the synthetic compounds

^{1}H NMR and ^{13}C NMR spectra for 7,8-dihydroxy-4-methyl-2*H*-chromen-2-one (2)

^{1}H NMR (600 MHz, DMSO-d_6)

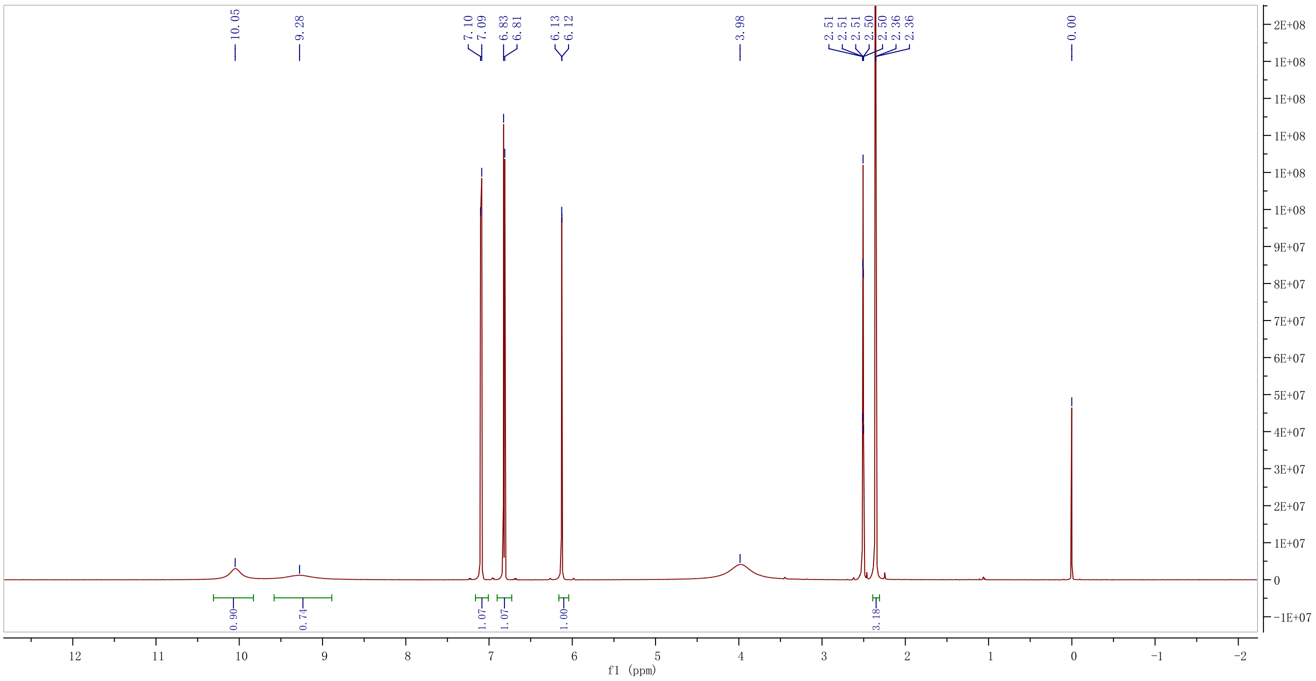

^{13}C NMR (151 MHz, DMSO-d_6)

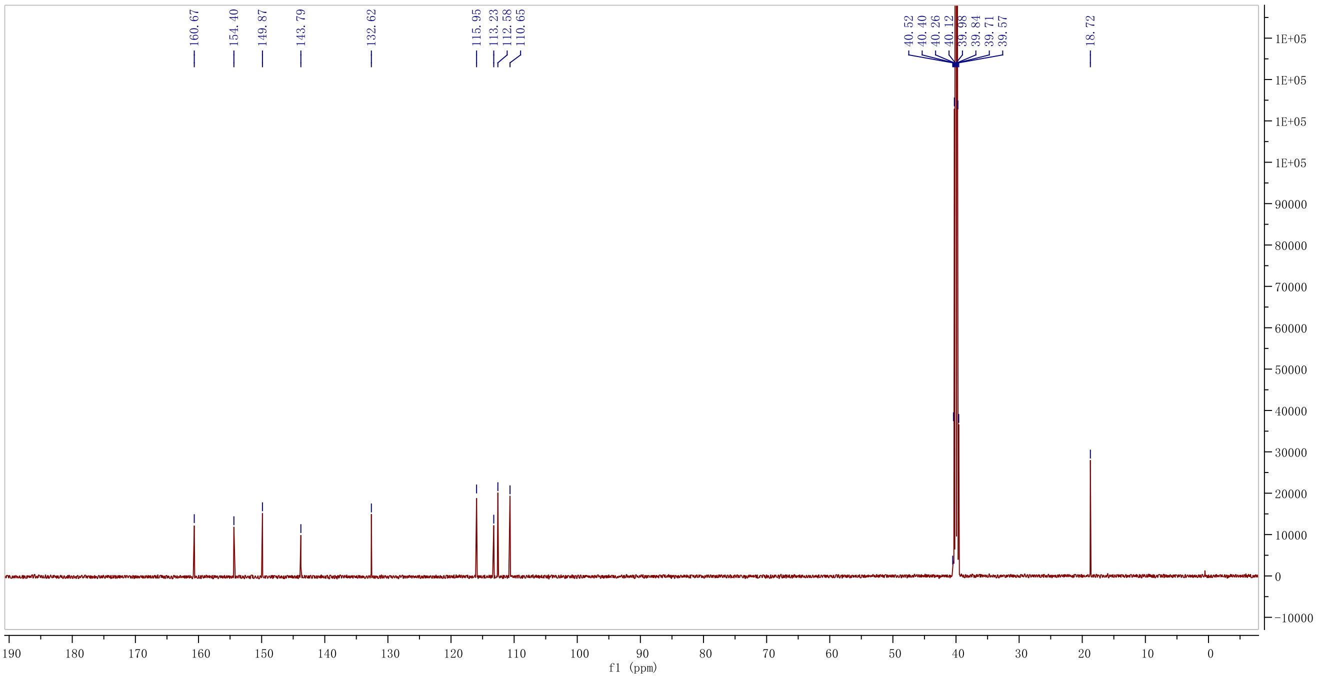

^{1}H NMR and ^{13}C NMR spectra for 7,8-dihydroxy-4-phenyl-2*H*-chromen-2-one (3)

^{1}H NMR (600 MHz, DMSO-d_6)

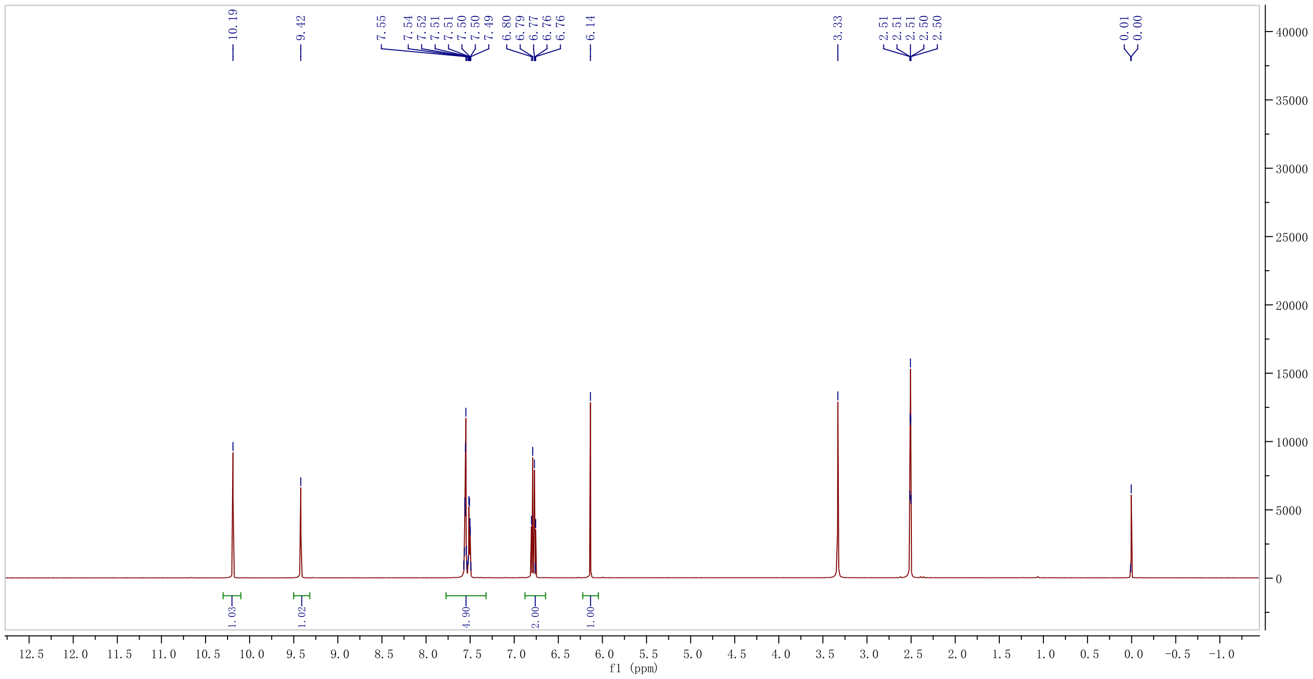

^{13}C NMR (151 MHz, DMSO-d_6)

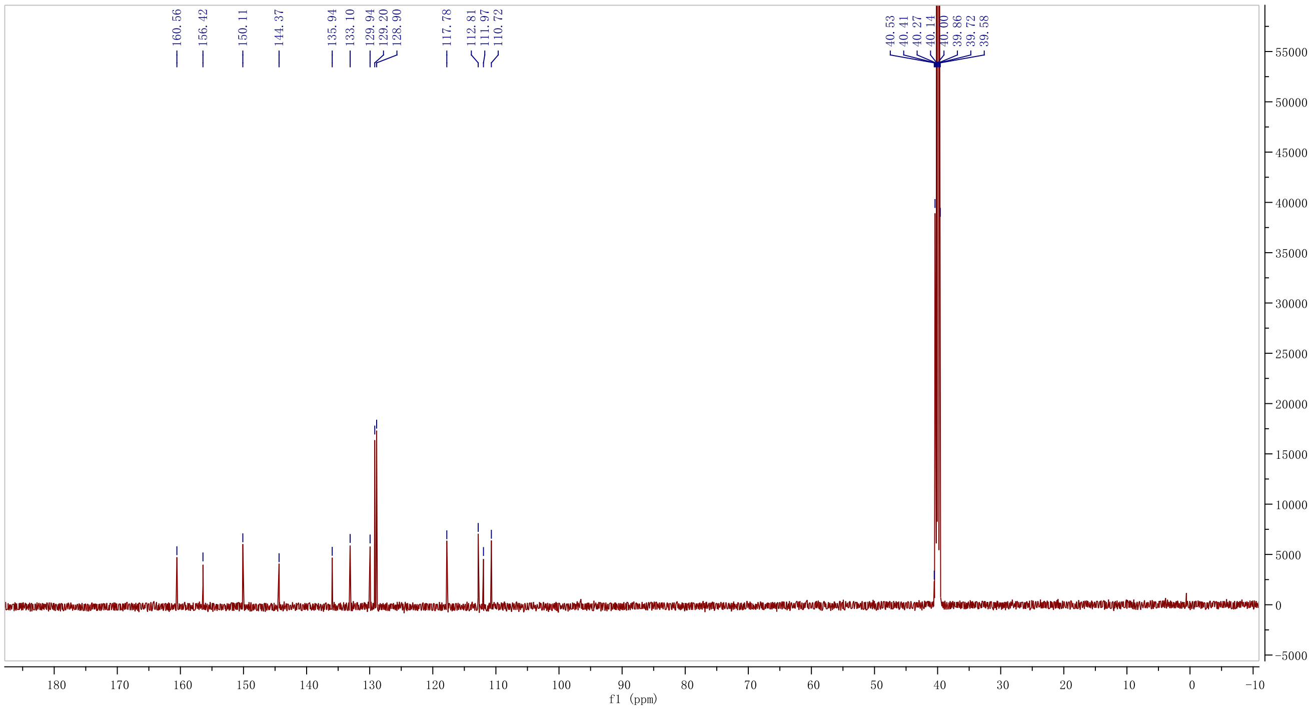

^{1}H NMR and ^{13}C NMR spectra for 7,8-dihydroxy-4- tert-butyl-2*H*-chromen-2-one (4)

^{1}H NMR (600 MHz, DMSO-d_6)

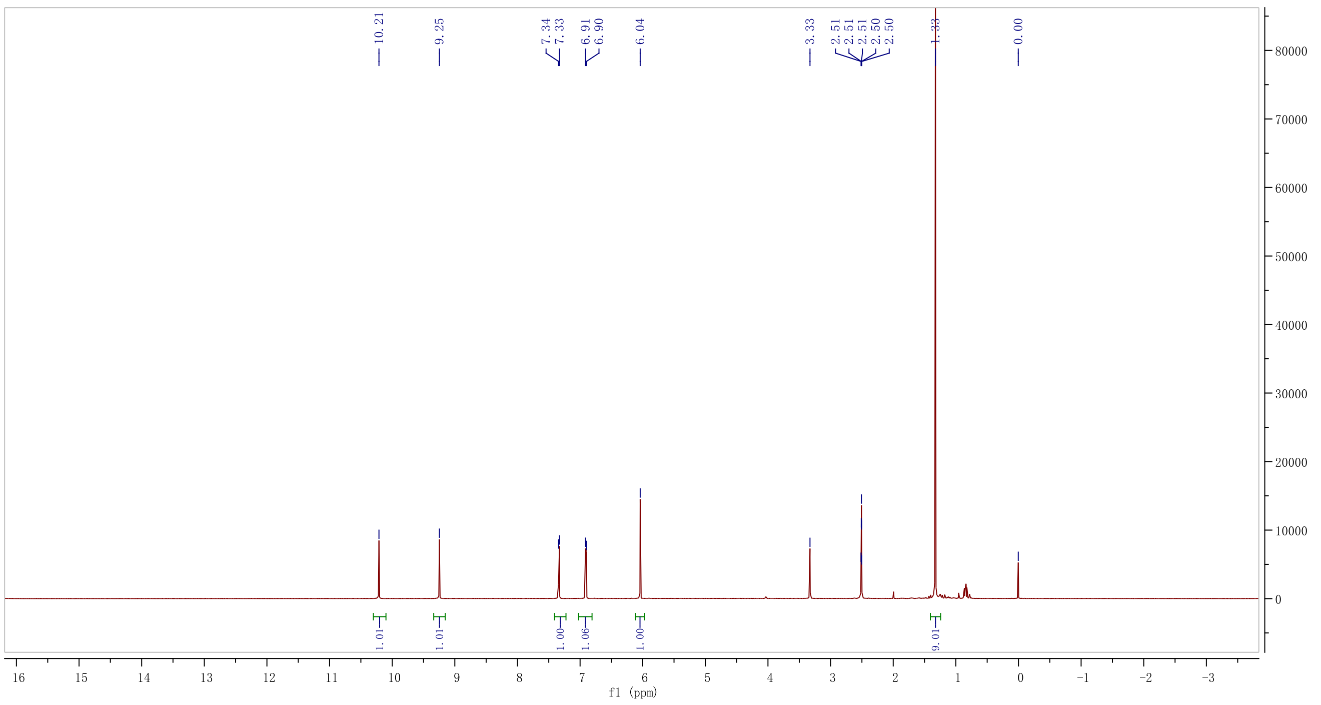

^{13}C NMR (151 MHz, DMSO-d_6)

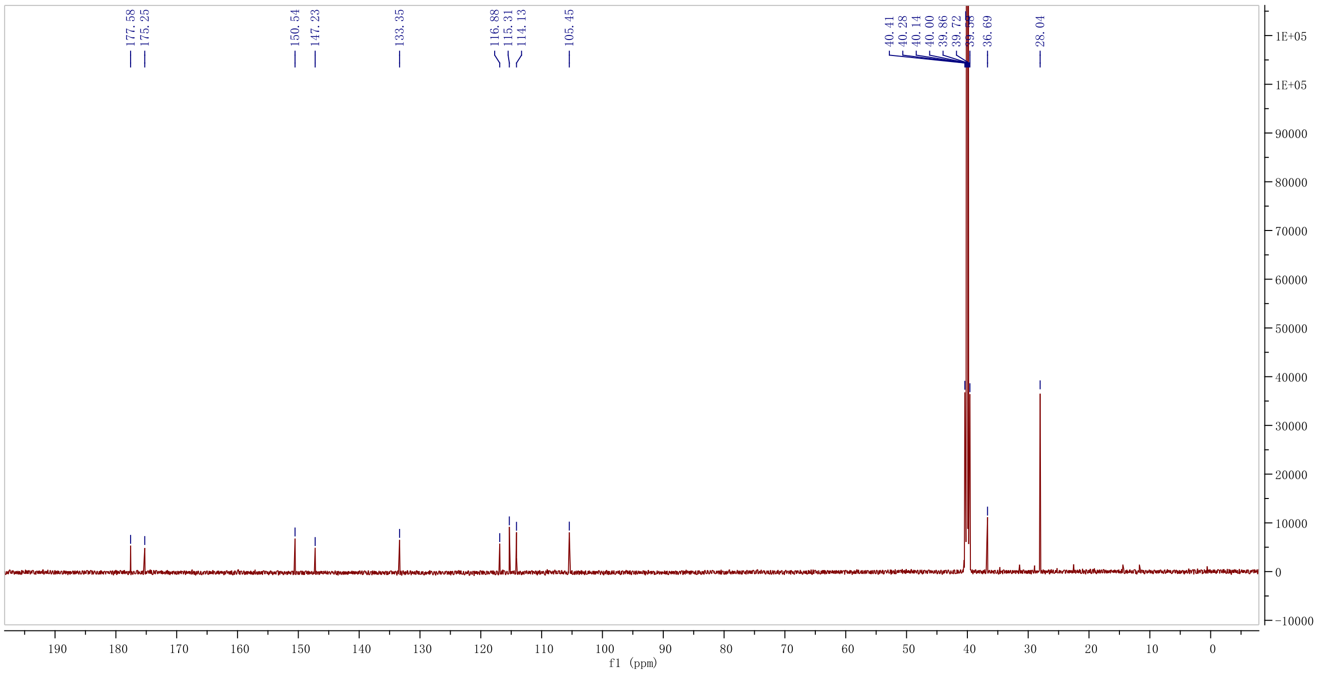

^{1}H NMR and ^{13}C NMR spectra for 6,7-dihydroxy-4-trifluoromethyl-2*H*-chromen-2-one (5)

^{1}H NMR (600 MHz, DMSO-d_6)

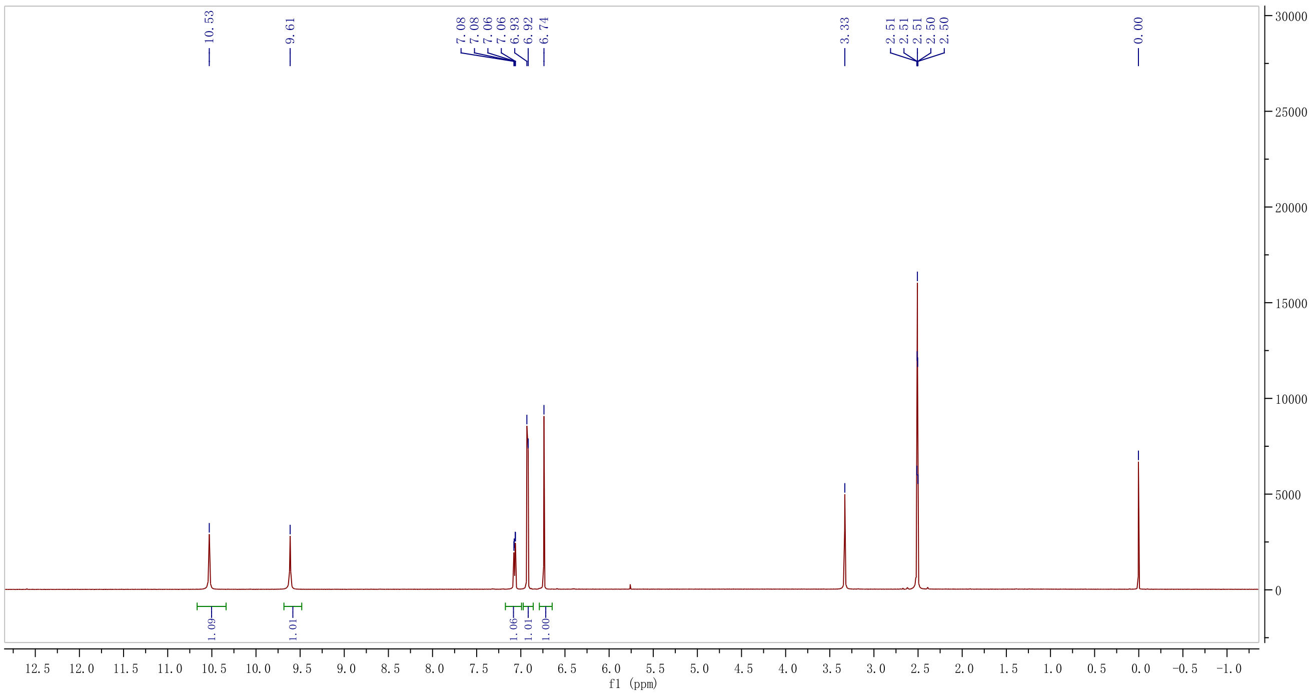

^{13}C NMR (151 MHz, DMSO-d_6)

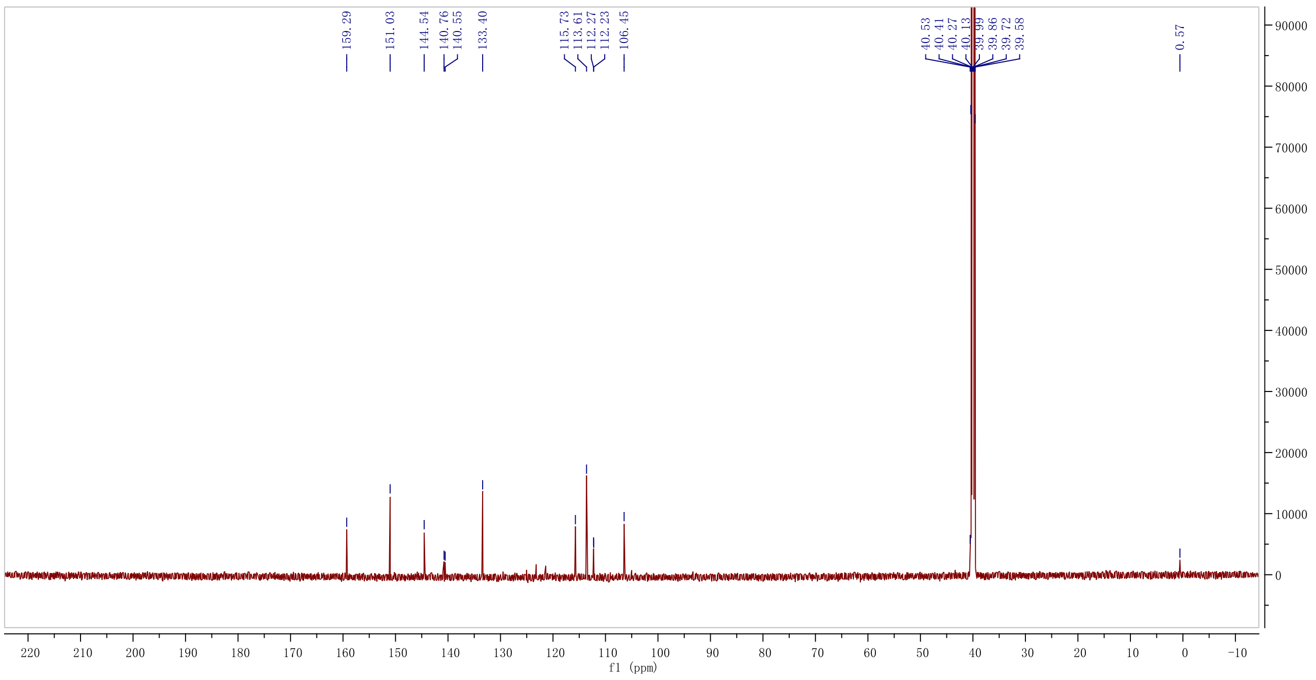

^{1}H NMR and ^{13}C NMR sprectra for 7,8-dihydroxy-4-chloromethyl-2*H*-chromen-2-one (6)

^{1}H NMR (600 MHz, DMSO-d_6)

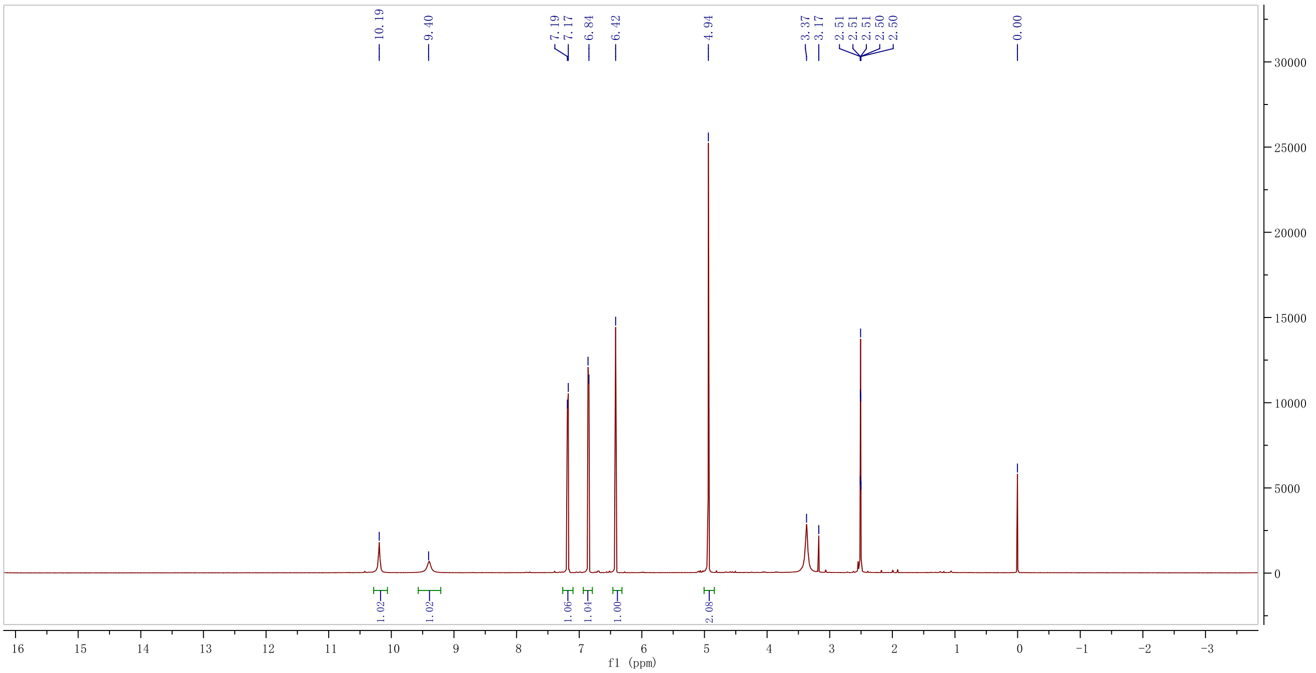

^{13}C NMR (151 MHz, DMSO-d_6)

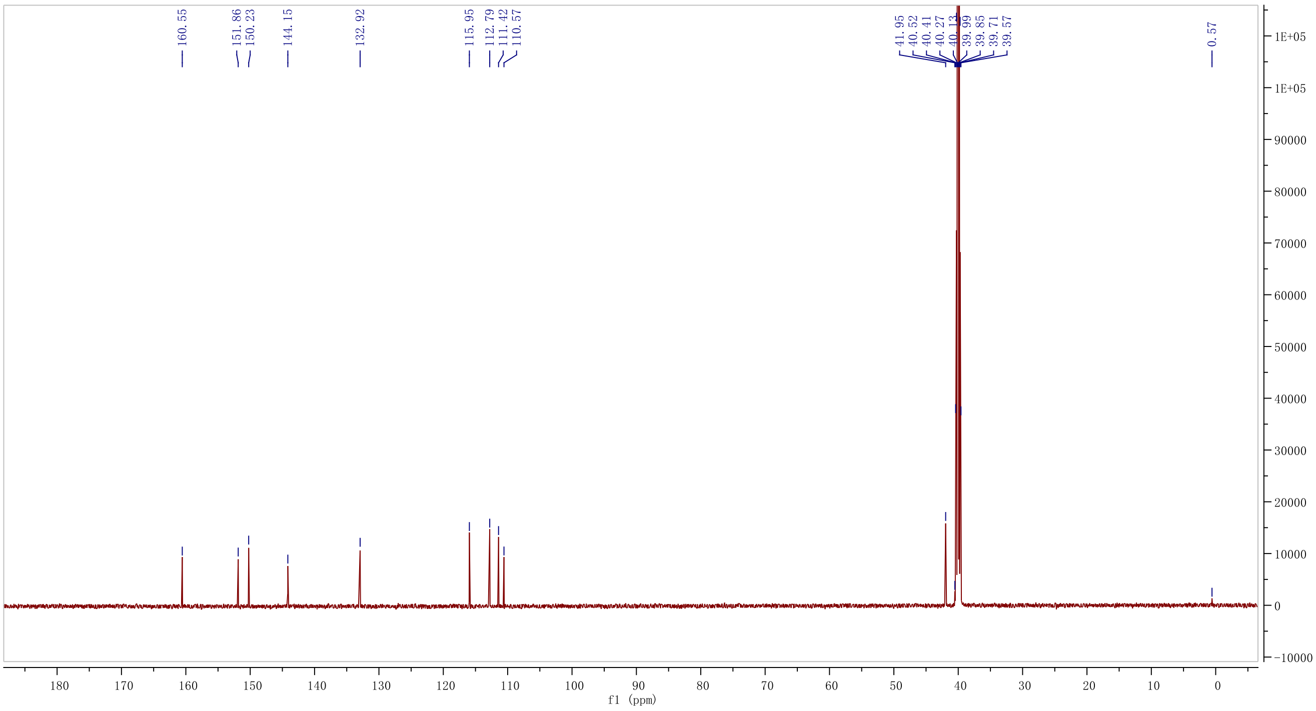

^{1}H NMR and ^{13}C NMR spectra for 7,8-dihydroxy-4-hydroxymethyl-2*H*-chromen-2-one (7)

^{1}H NMR (600 MHz, DMSO-d_6)

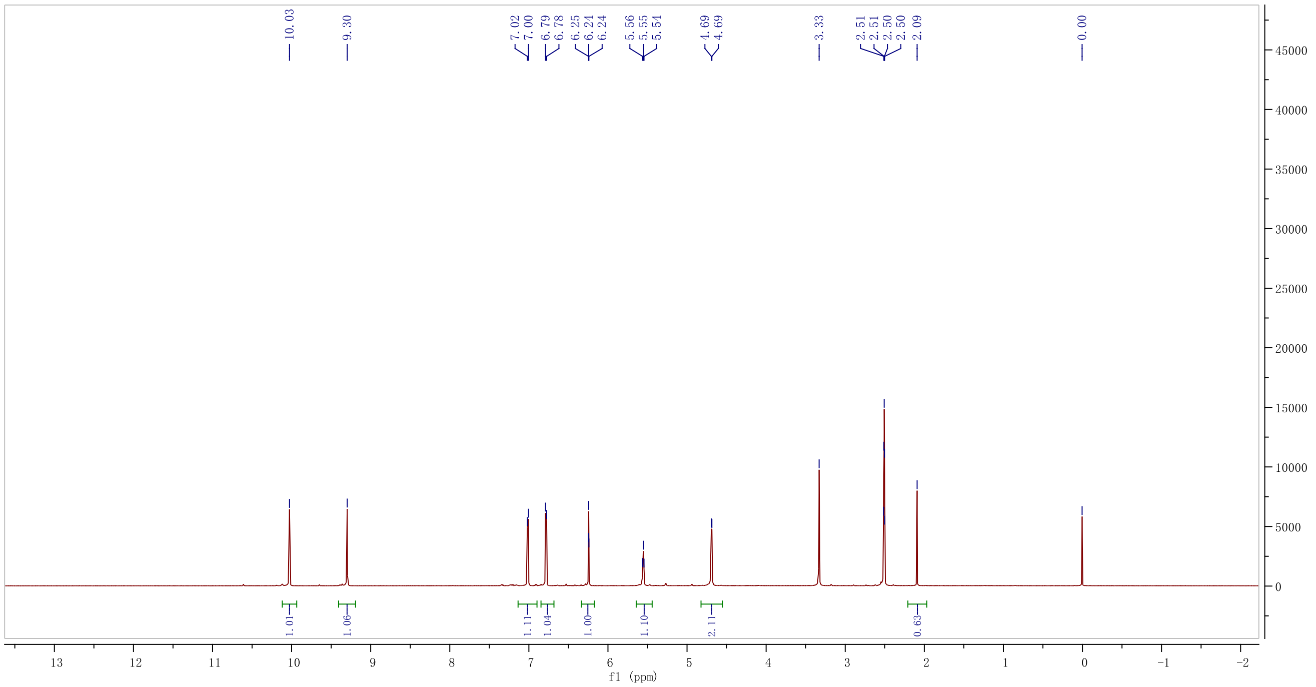

^{13}C NMR (151 MHz, DMSO-d_6)

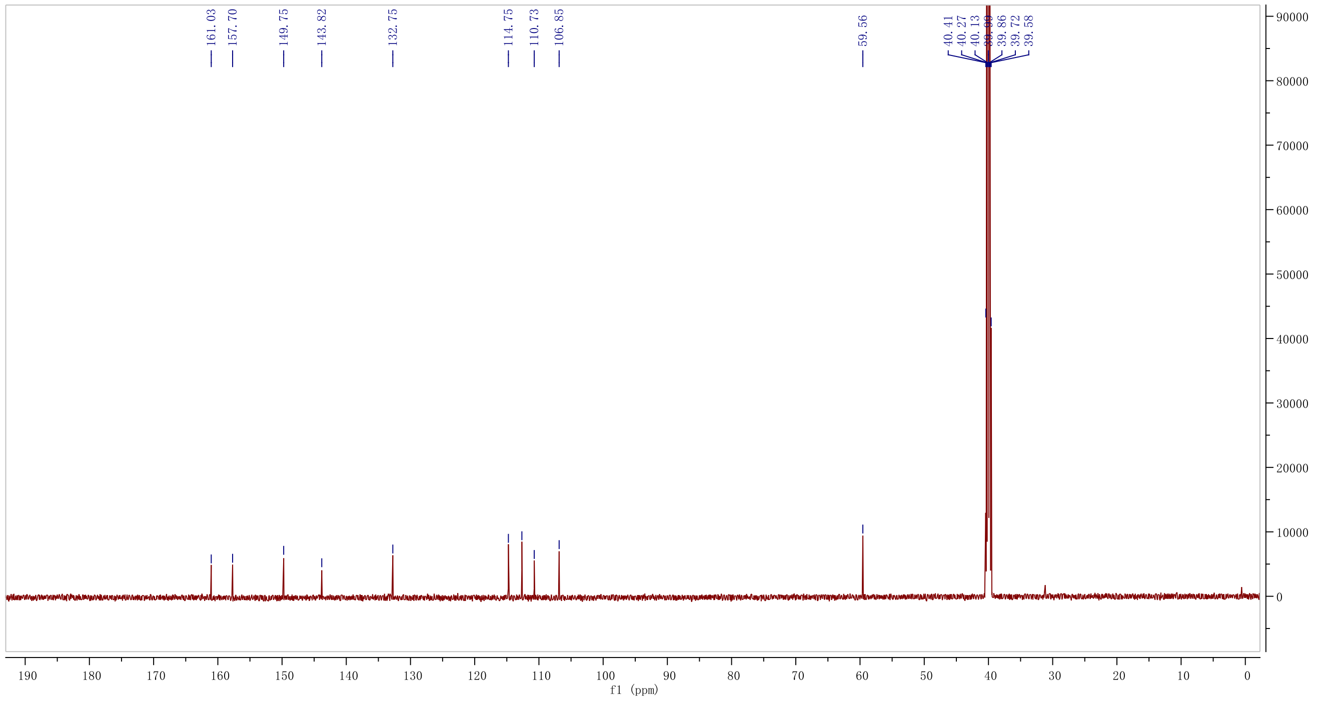

^{1}H NMR and ^{13}C NMR spectra for 7,8-dihydroxy-4-azidomethyl-2*H*-chromen-2-one (8)

^{1}H NMR (600 MHz, DMSO-d_6)

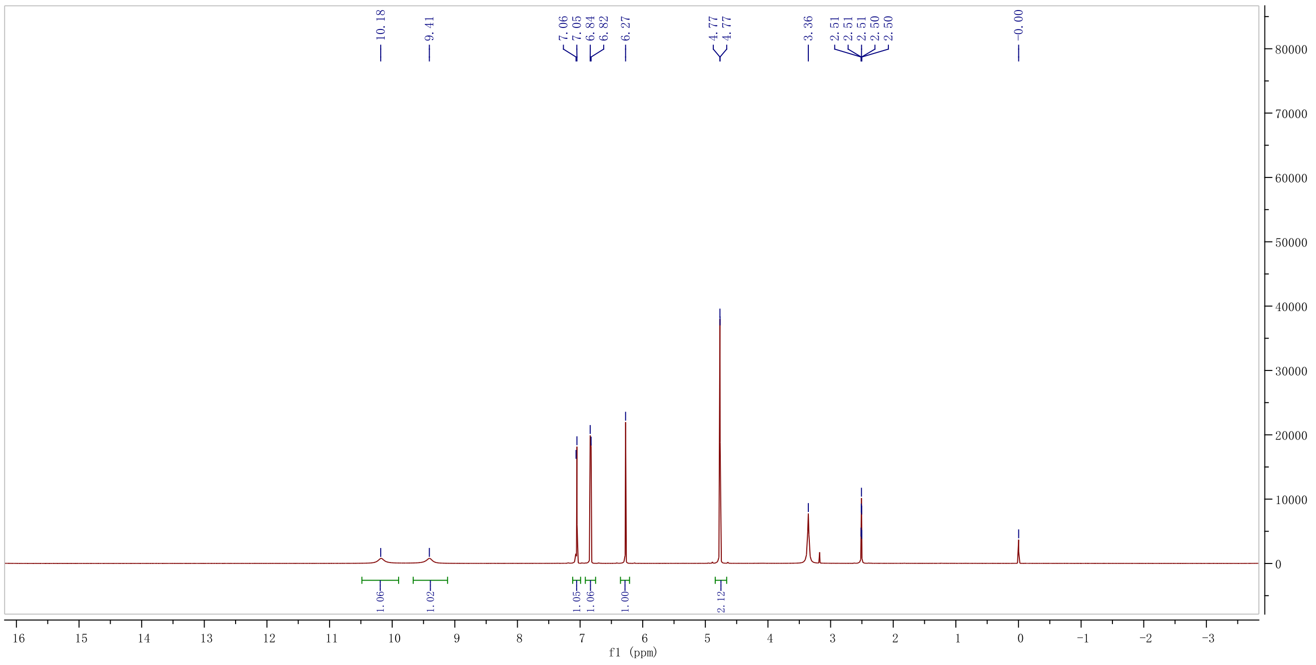

^{13}C NMR (151 MHz, DMSO-d_6)

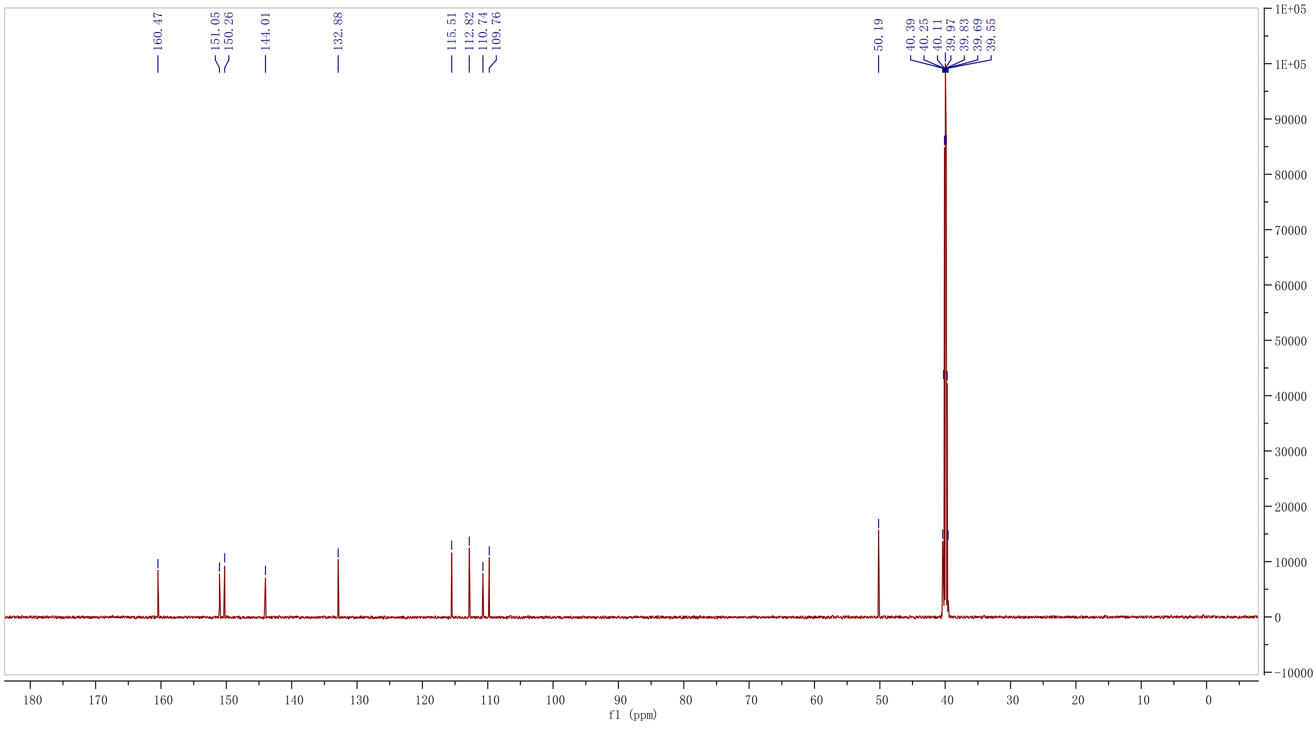

^1H NMR and ^{13}C NMR spectra for 2-(7,8-dihydroxy-2-oxo-2*H*-chromen-4-yl)acetic acid (9)

^1H NMR (400 MHz, DMSO-d_6)

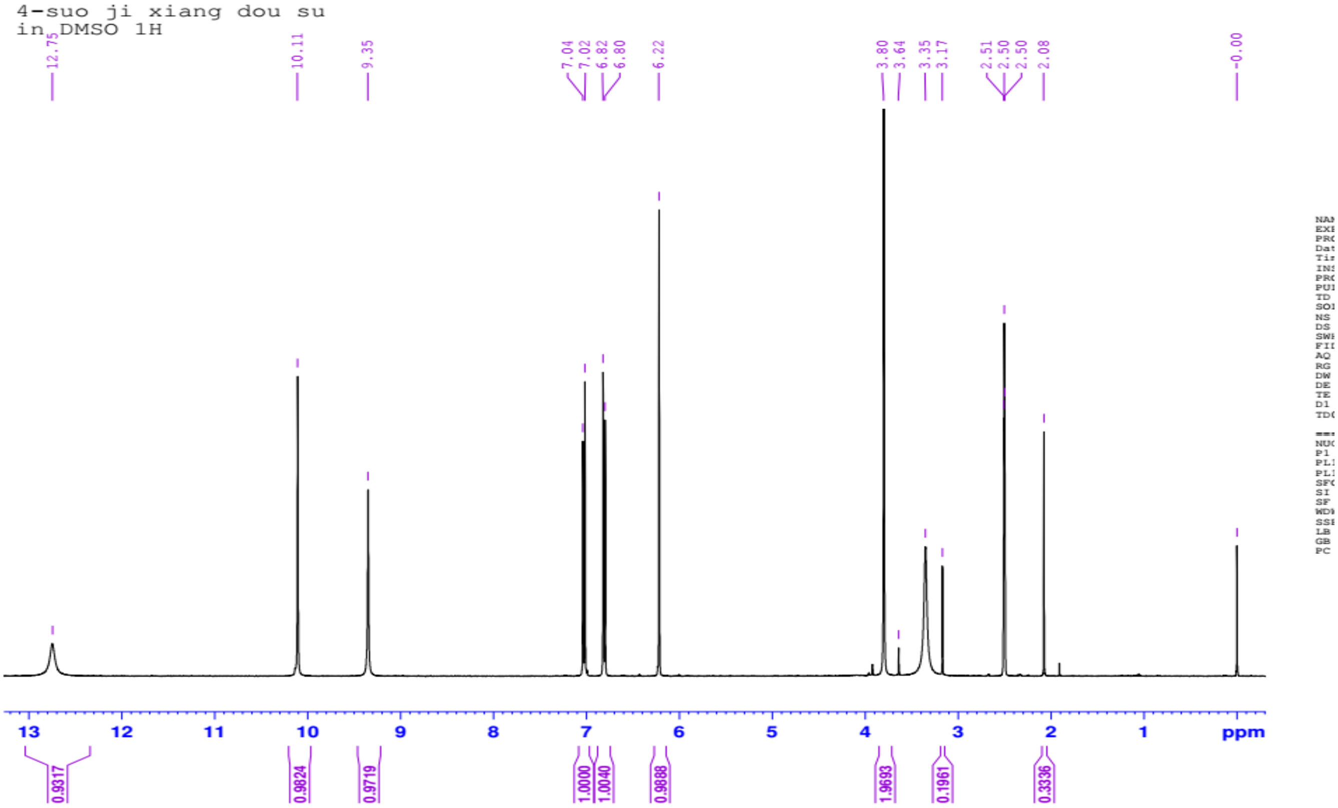

^{13}C NMR (100 MHz, DMSO-d_6)

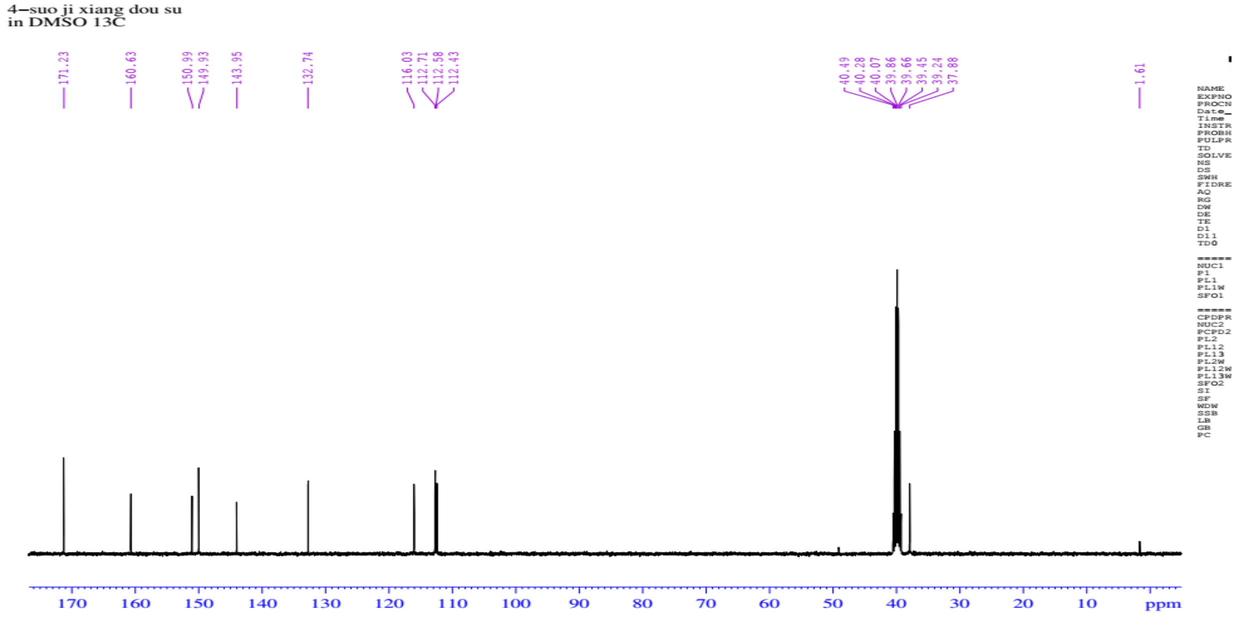

^{1}H NMR and ^{13}C NMR spectra for methyl 2-(7,8-dihydroxy-2-oxo-2*H*-chromen-4-yl)acetic acid (10)

^{1}H NMR (600 MHz, DMSO-d_6)

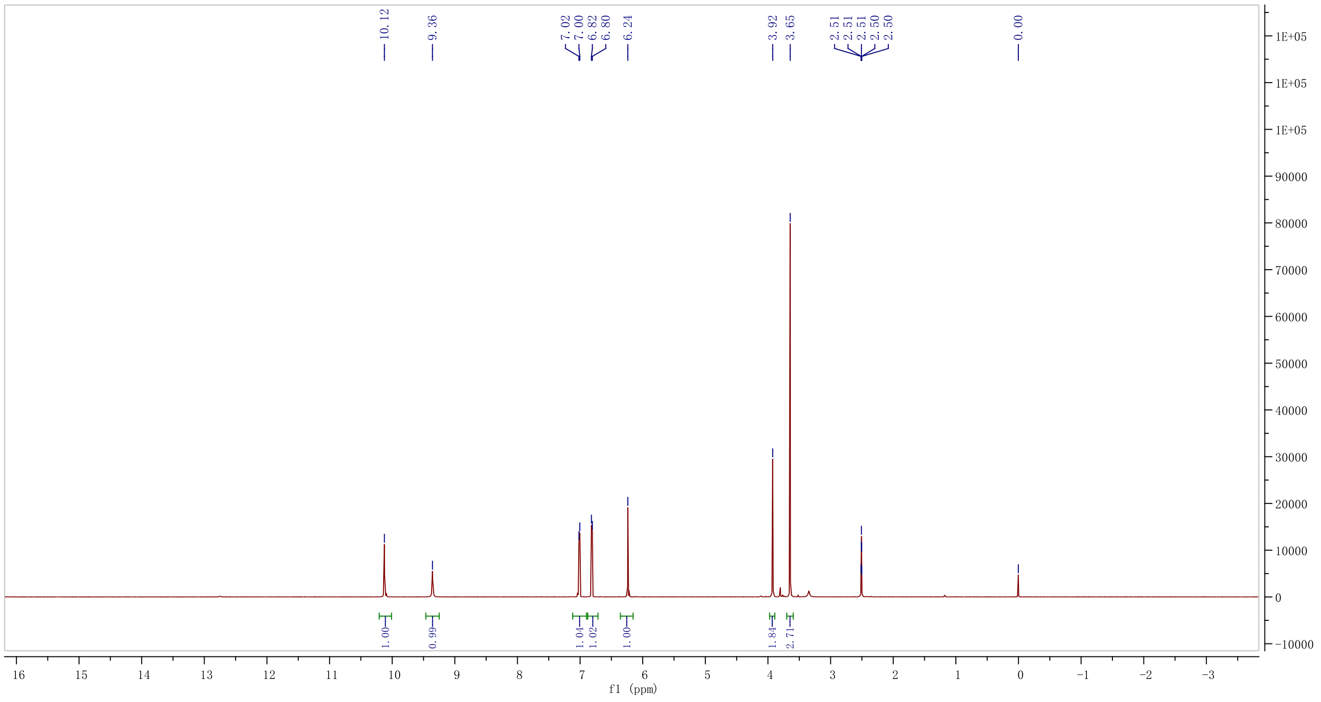

^{13}C NMR (151 MHz, DMSO-d_6)

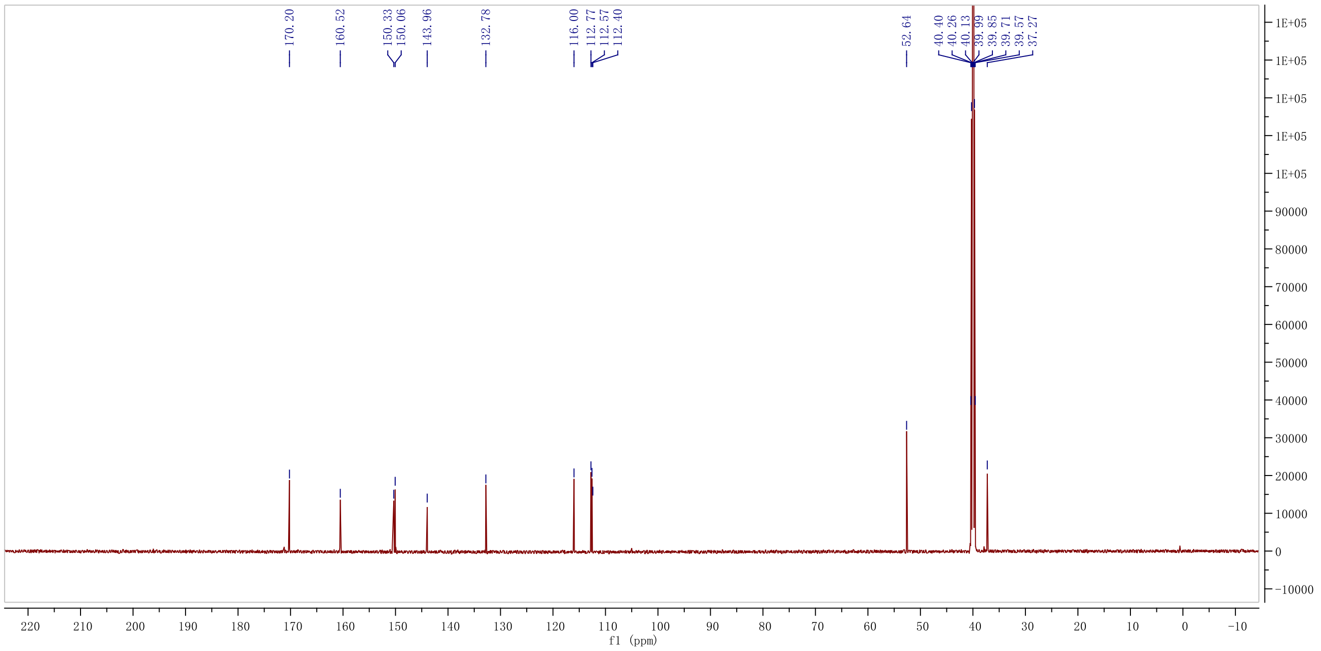

^{1}H NMR and ^{13}C NMR spectra for 2-oxo-2*H*-chromene-7,8-diyl diacetate (12)

^{1}H NMR (400 MHz, DMSO-d_6)

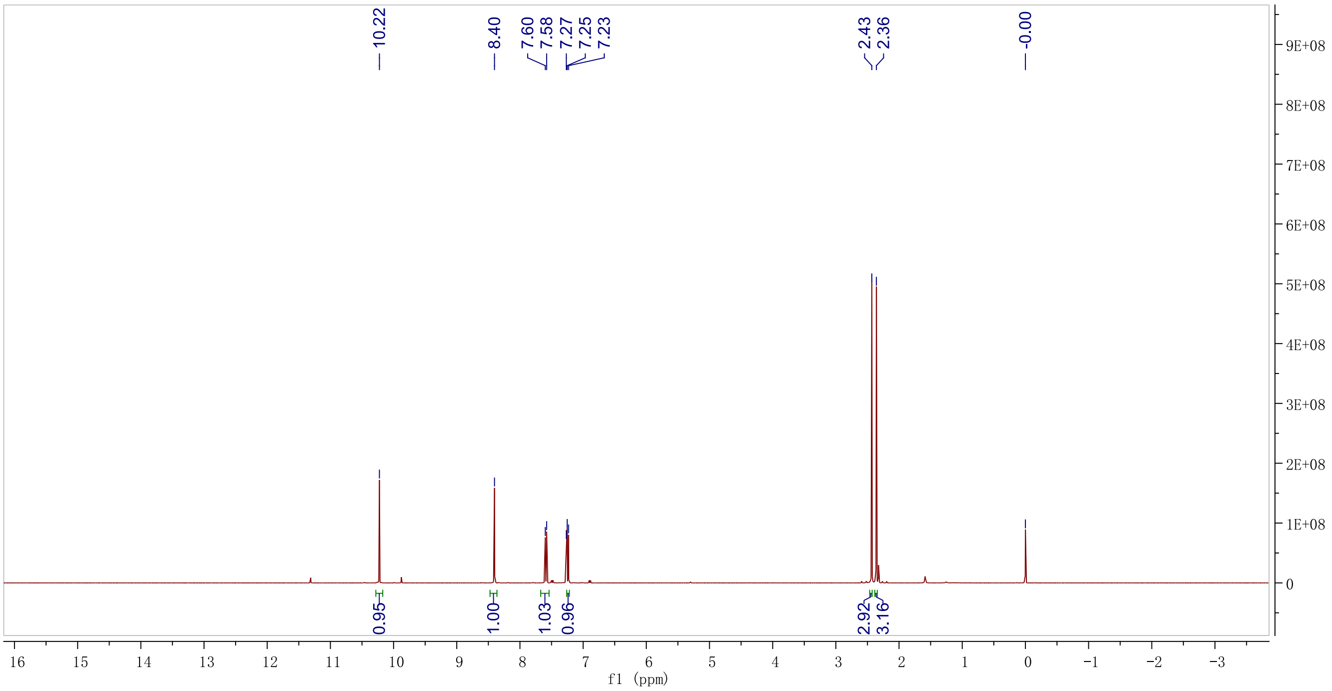

^{13}C NMR (100 MHz, DMSO-d_6)

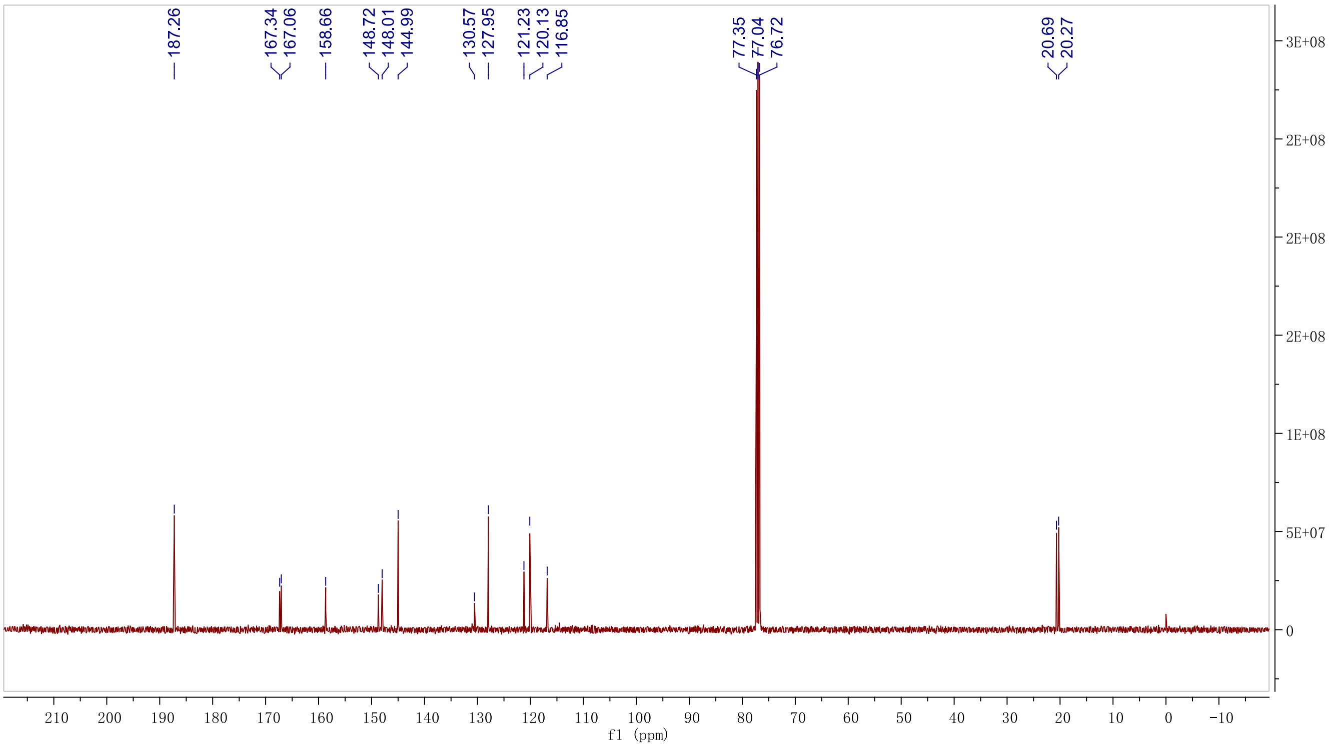

^{1}H NMR and ^{13}C NMR spectra for 7,8-dihydroxy-3-phenyl-2*H*-chromen-2-one (13)

^{1}H NMR (400 MHz, DMSO-d_6)

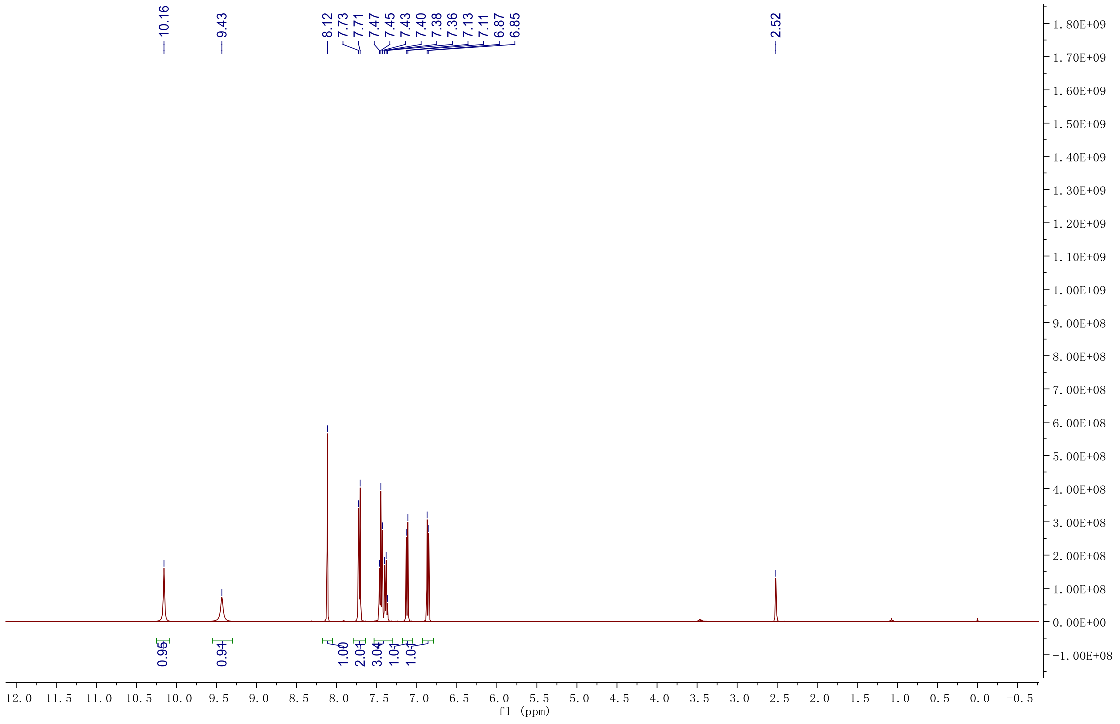

^{13}C NMR (100 MHz, DMSO-d_6)

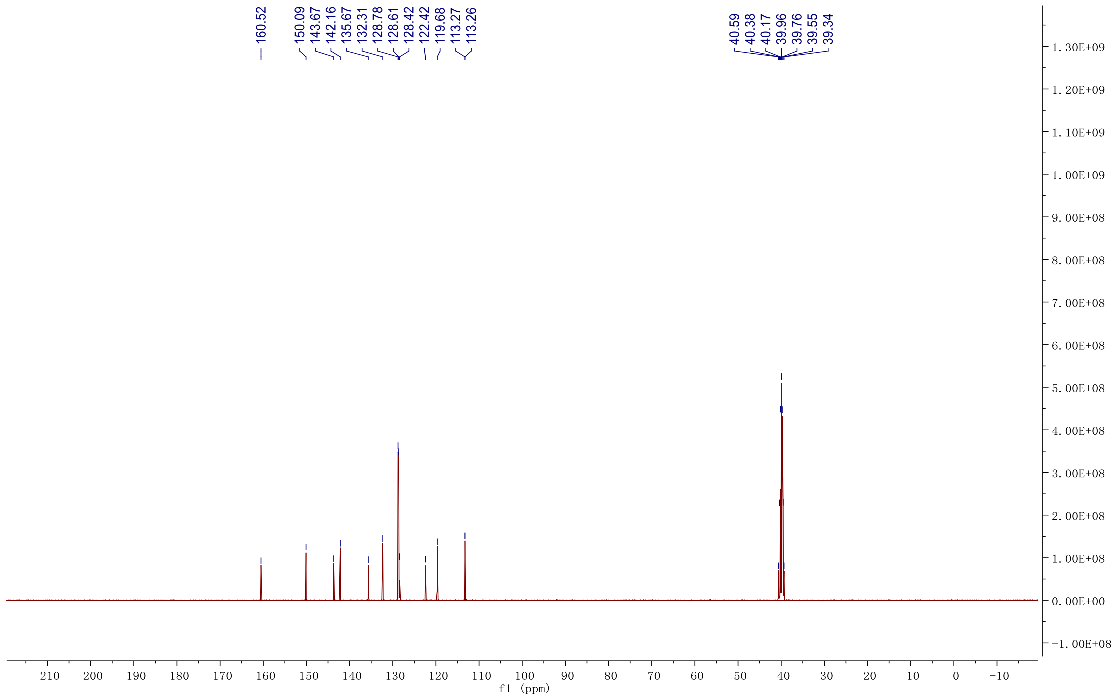

^{1}H NMR and ^{13}C NMR spectra for 7,8-dihydroxy-2-oxo-2*H*-chromene-3-carbonitrile（14）

^{1}H NMR (600 MHz, DMSO-d_6)

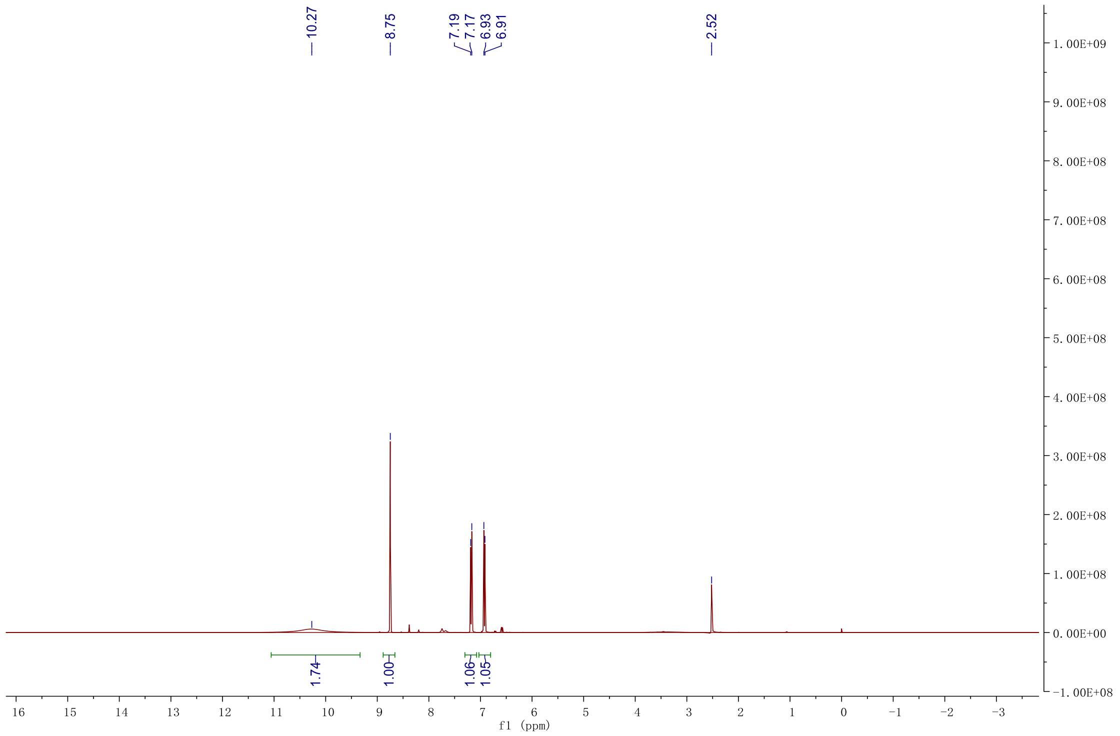

^{13}C NMR (151MHz, DMSO-d_6)

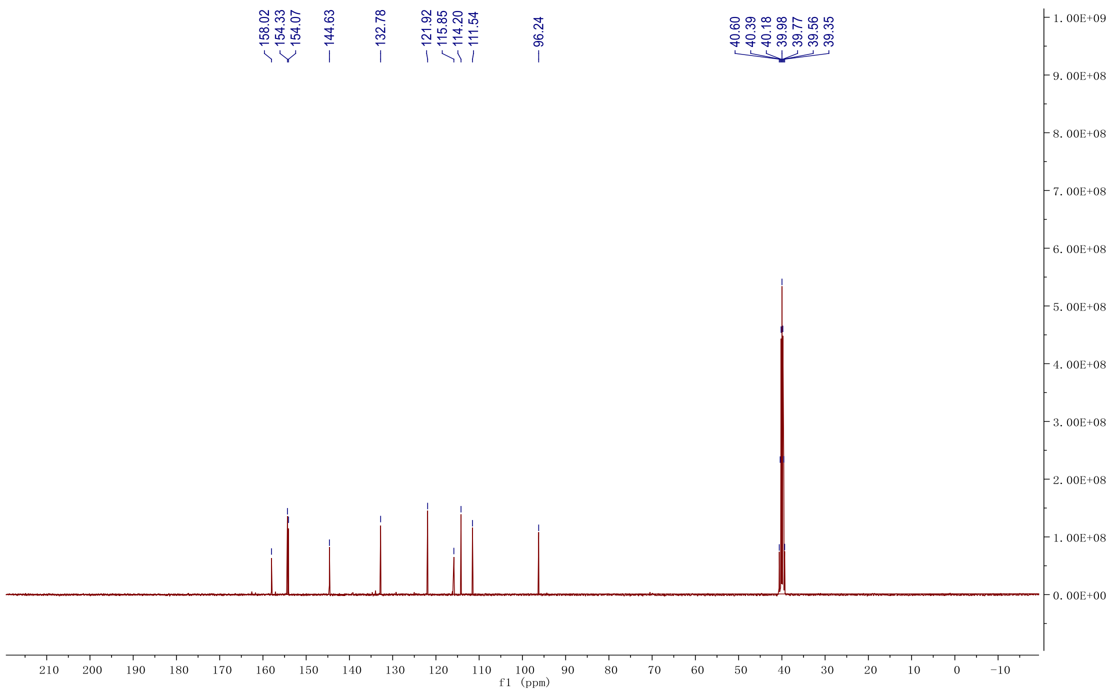

^{1}H NMR and ^{13}C NMR spectra for 3-acetyl-7,8-dihydroxy-2*H*-chromen-2-one (15)

^{1}H NMR (600 MHz, DMSO-d_6)

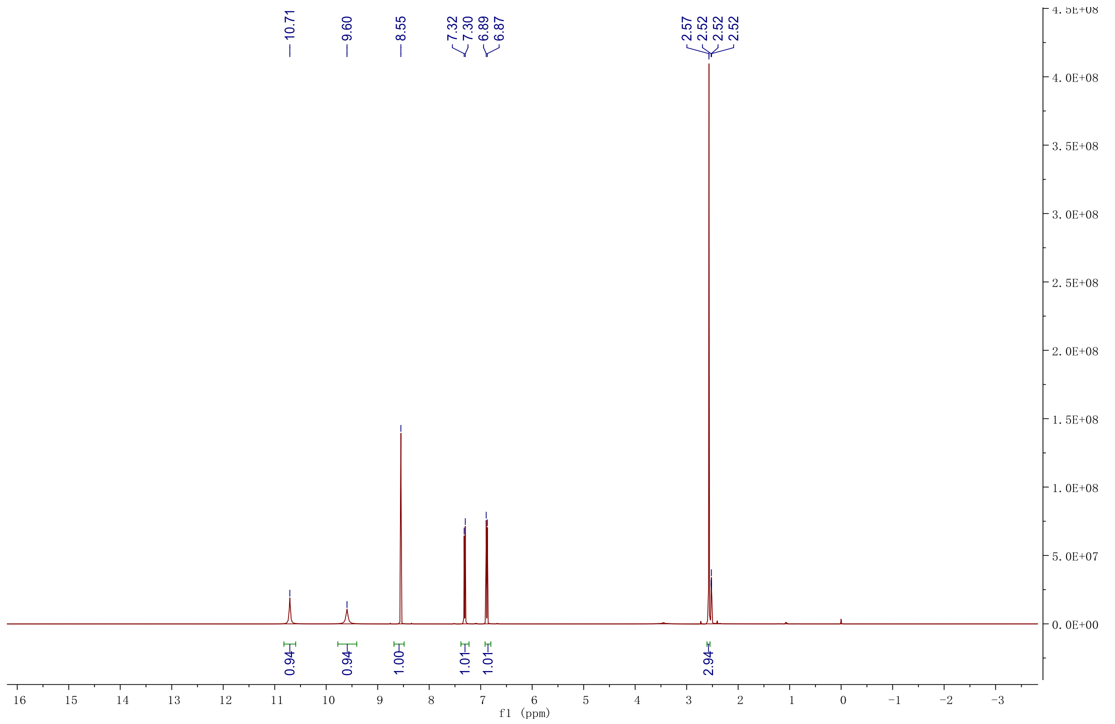

^{13}C NMR (151MHz, DMSO-d_6)

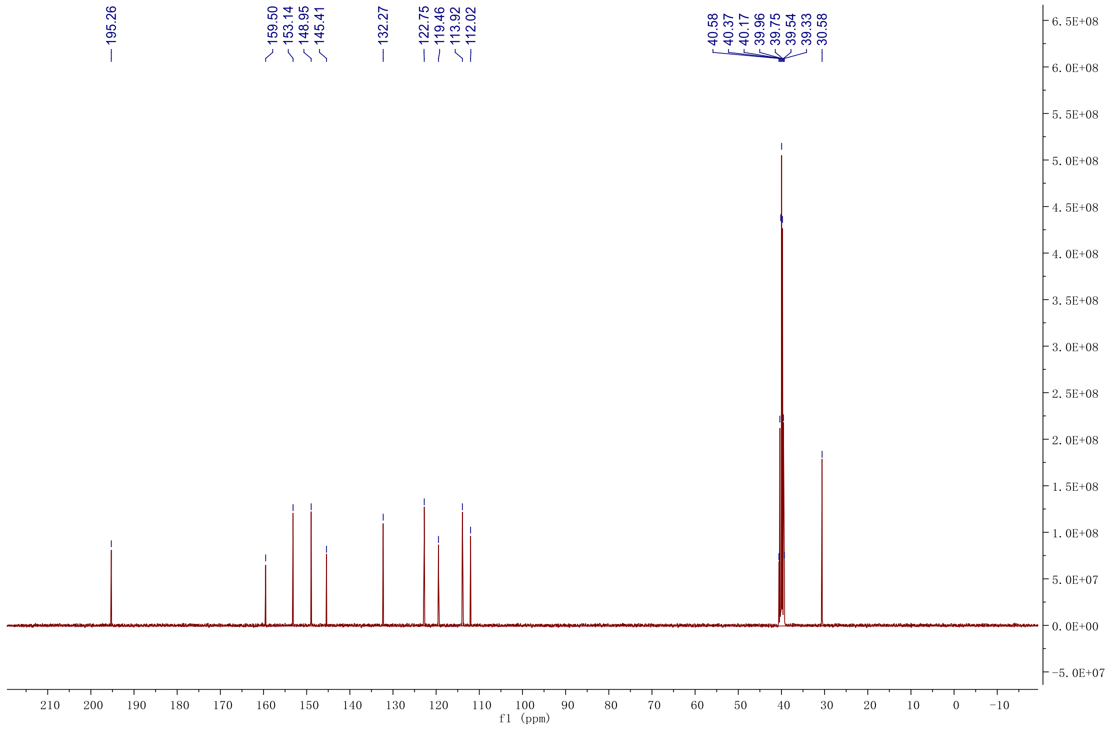

^{1}H NMR and ^{13}C NMR spectra for 7,8-dihydroxy-2-oxo-2*H*-chromene-3-carboxylic acid (16)

^{1}H NMR (600 MHz, DMSO-d_6)

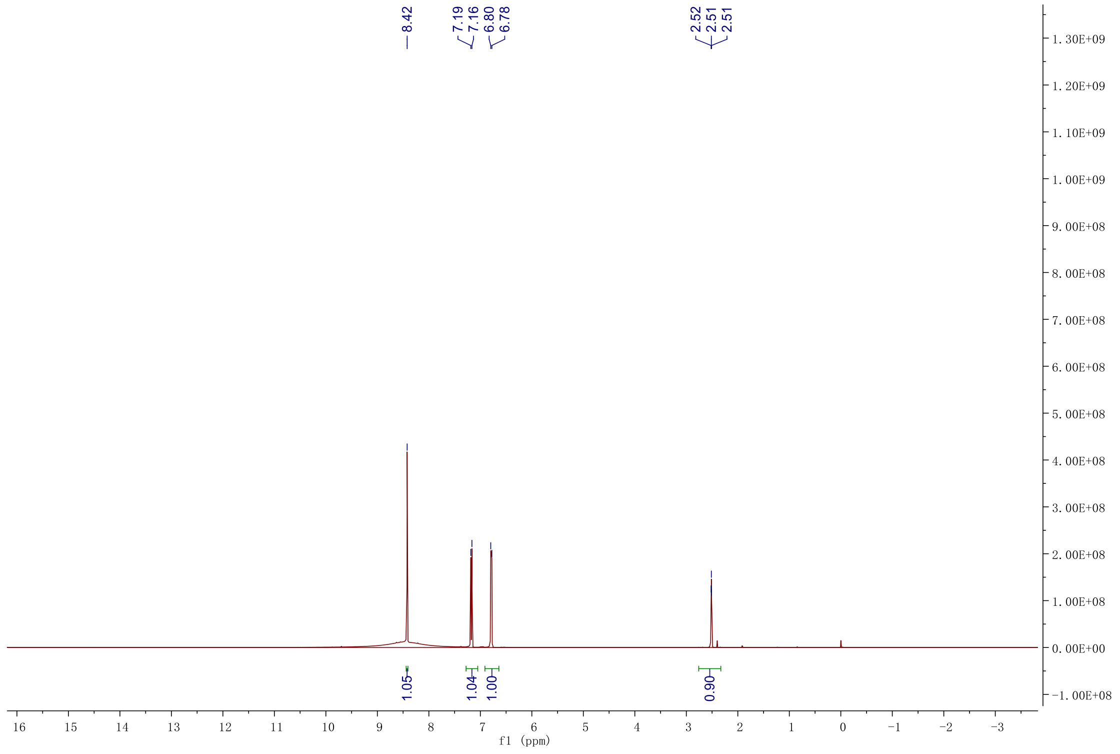

^{13}C NMR (151MHz, DMSO-d_6)

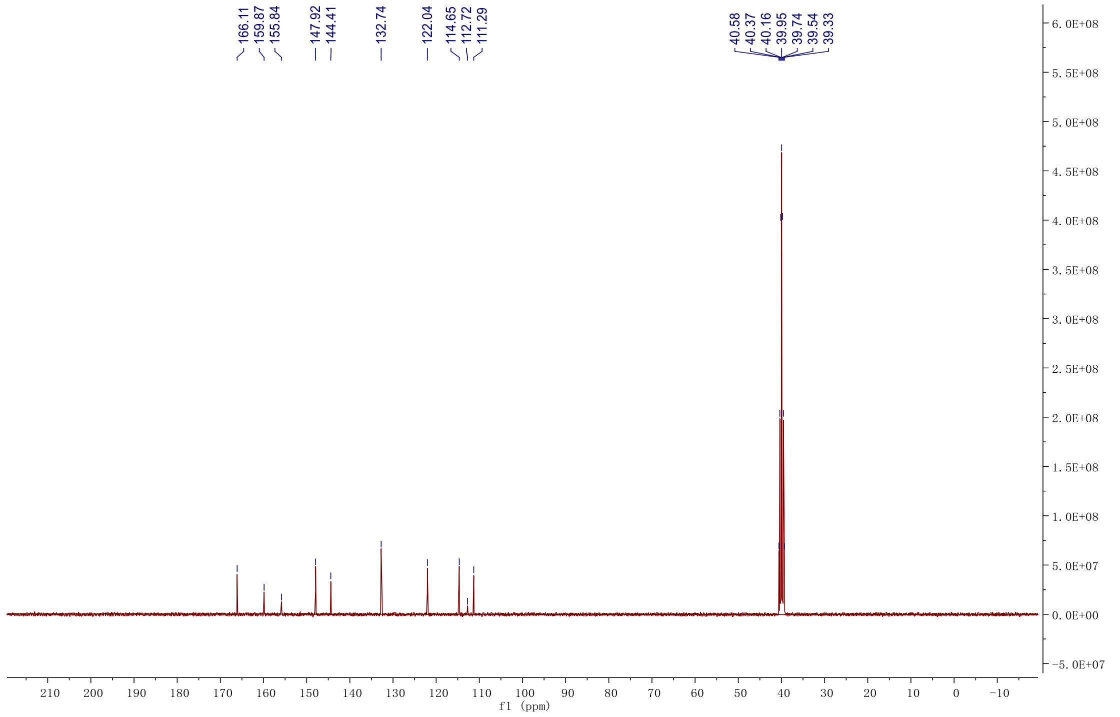

^{1}H NMR and ^{13}C NMR spectra for Ethyl 7,8-dihydroxy-2-oxo-2*H*-chromene-3-carboxylate (17)

^{1}H NMR (600 MHz, DMSO-d_6)

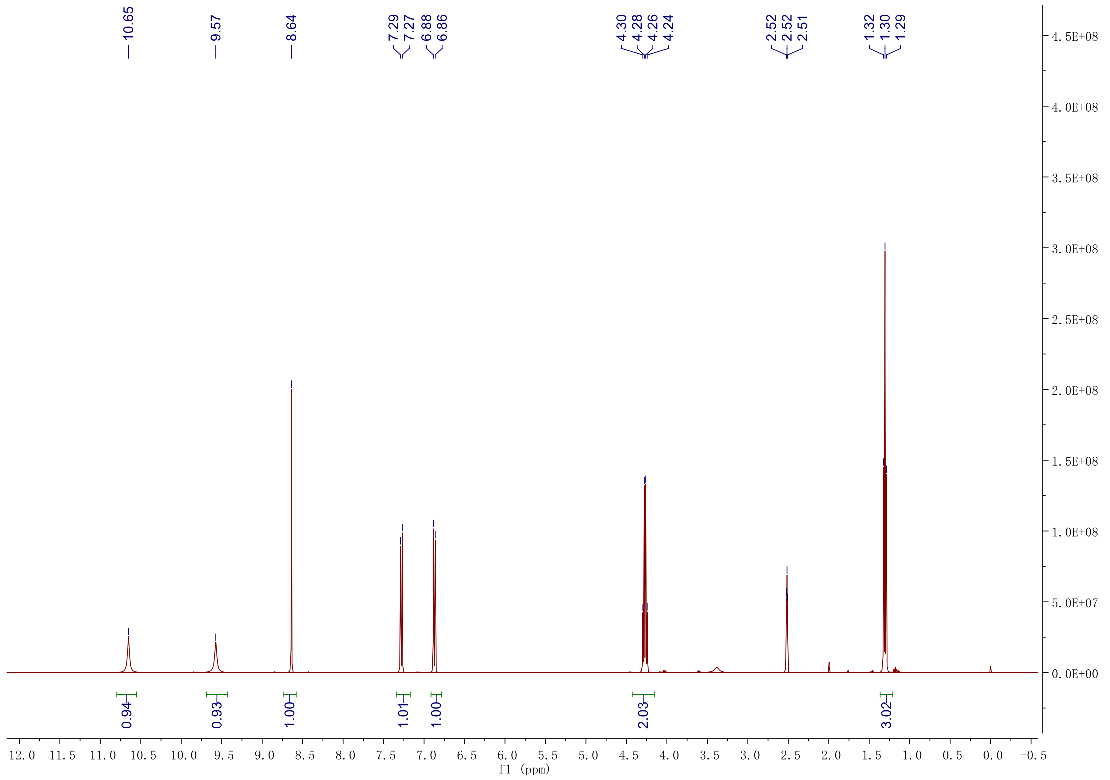

^{13}C NMR (151MHz, DMSO-d_6)

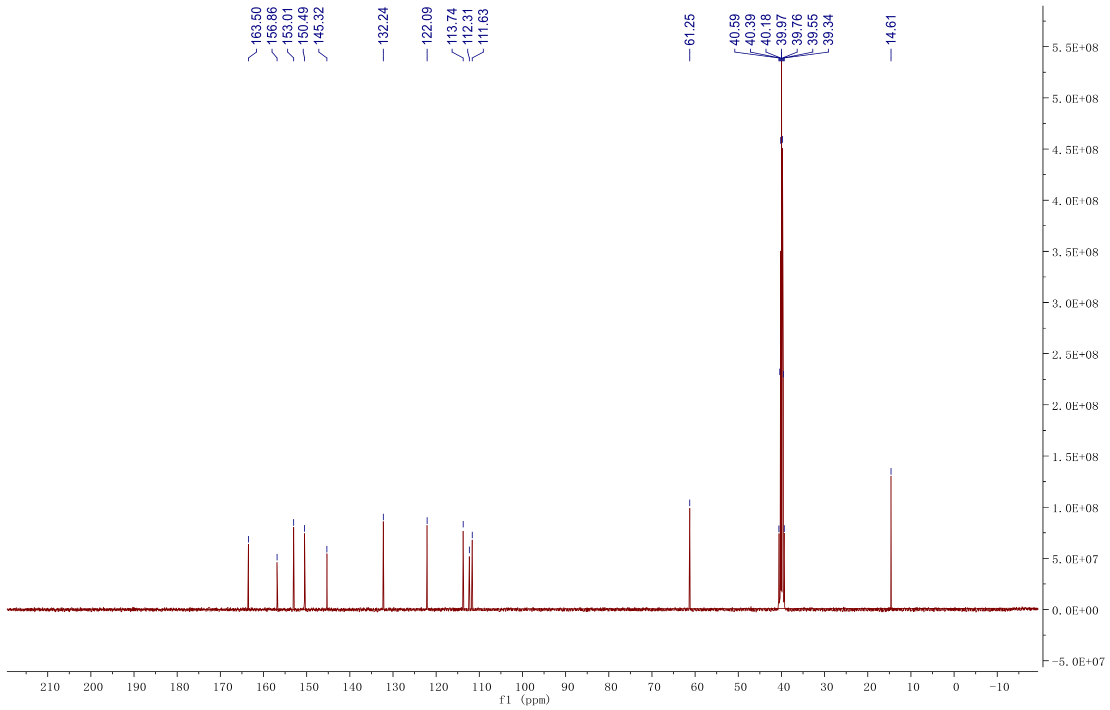

^{1}H NMR and ^{13}C NMR spectra for 3-(benzo[d]thiazol-2-yl)-7,8-dihydroxy-2*H*-chromen-2-one (19)

^{1}H NMR (600 MHz, DMSO-d_6)

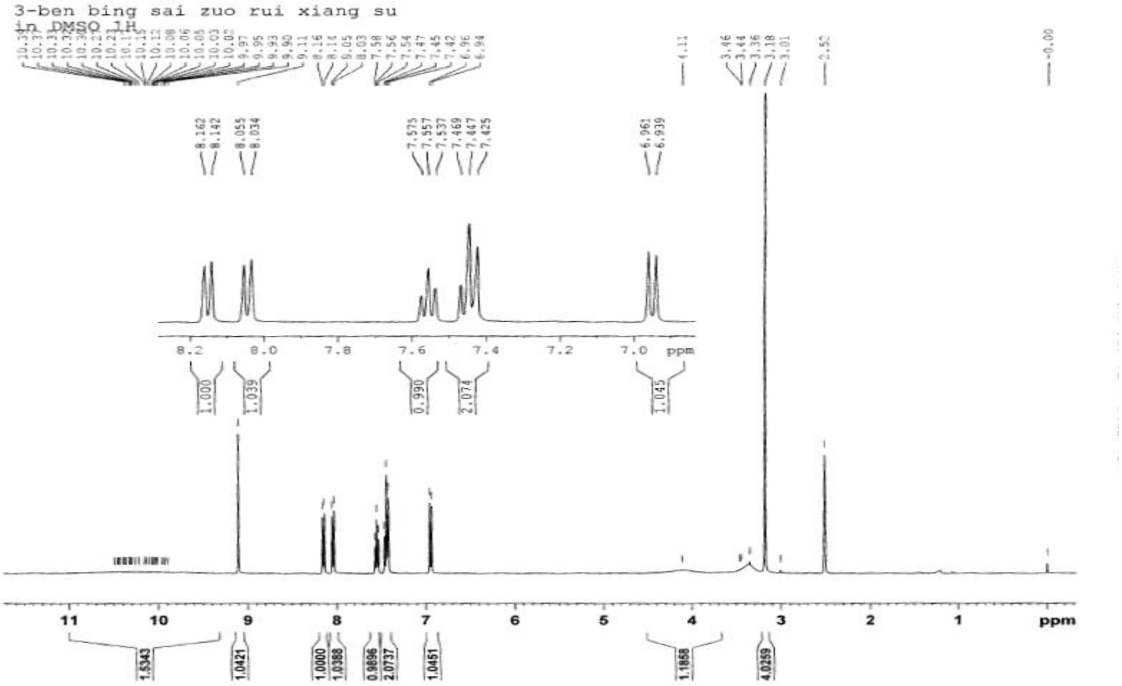

^{13}C NMR (100MHz, DMSO-d_6)

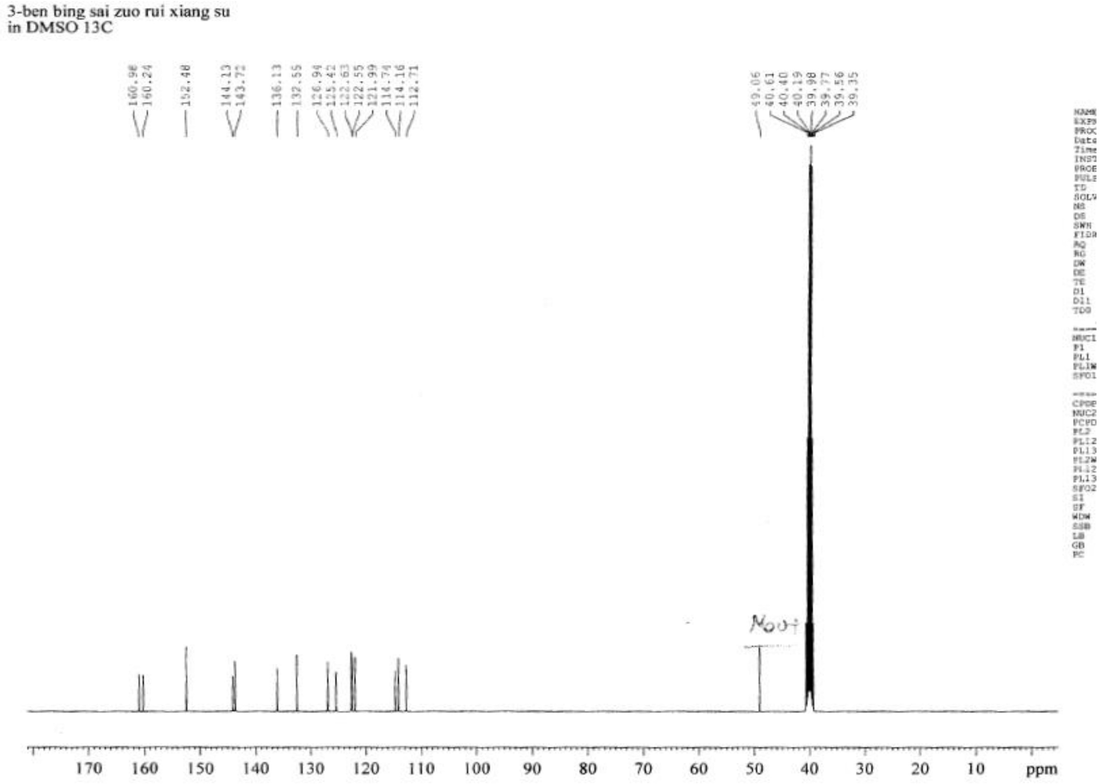

^{1}H NMR and ^{13}C NMR spectra for 3-(benzo[*d*]thiazol-2-yl)-7,8-dihydroxy-2-oxo-2*H*-chromene-4-carboxylic acid (21)

^{1}H NMR (600 MHz, DMSO-d_6)

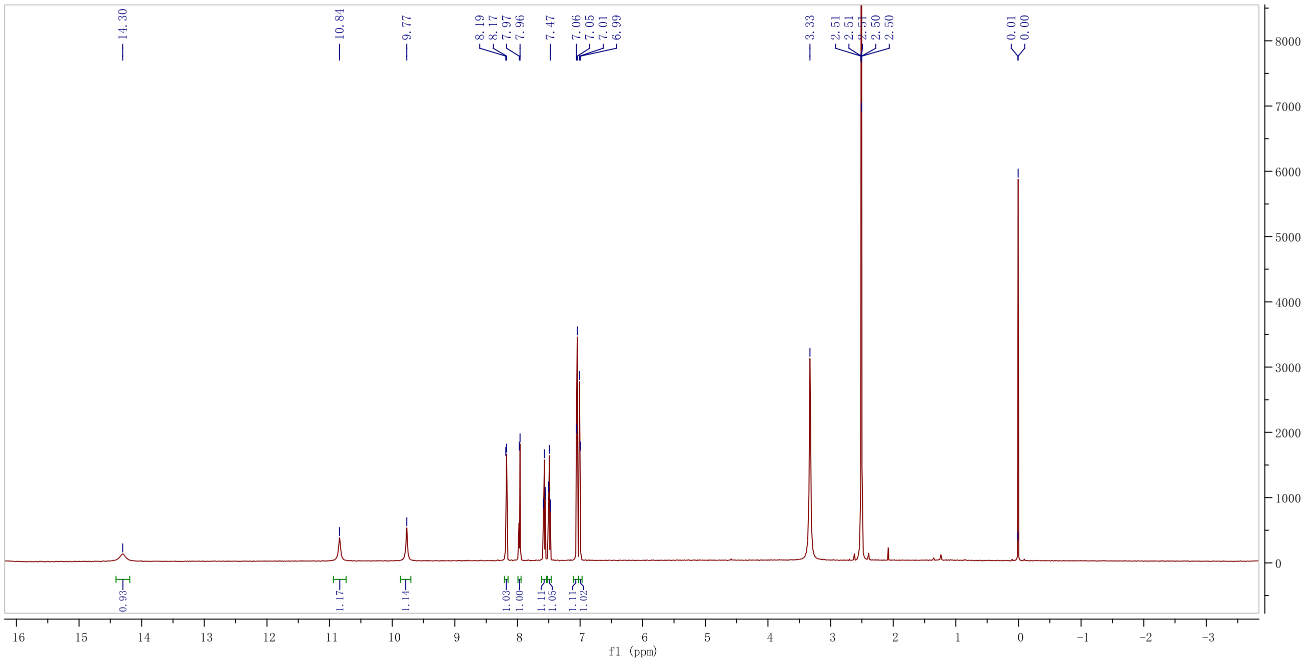

^{13}C NMR (151MHz, DMSO-d_6)

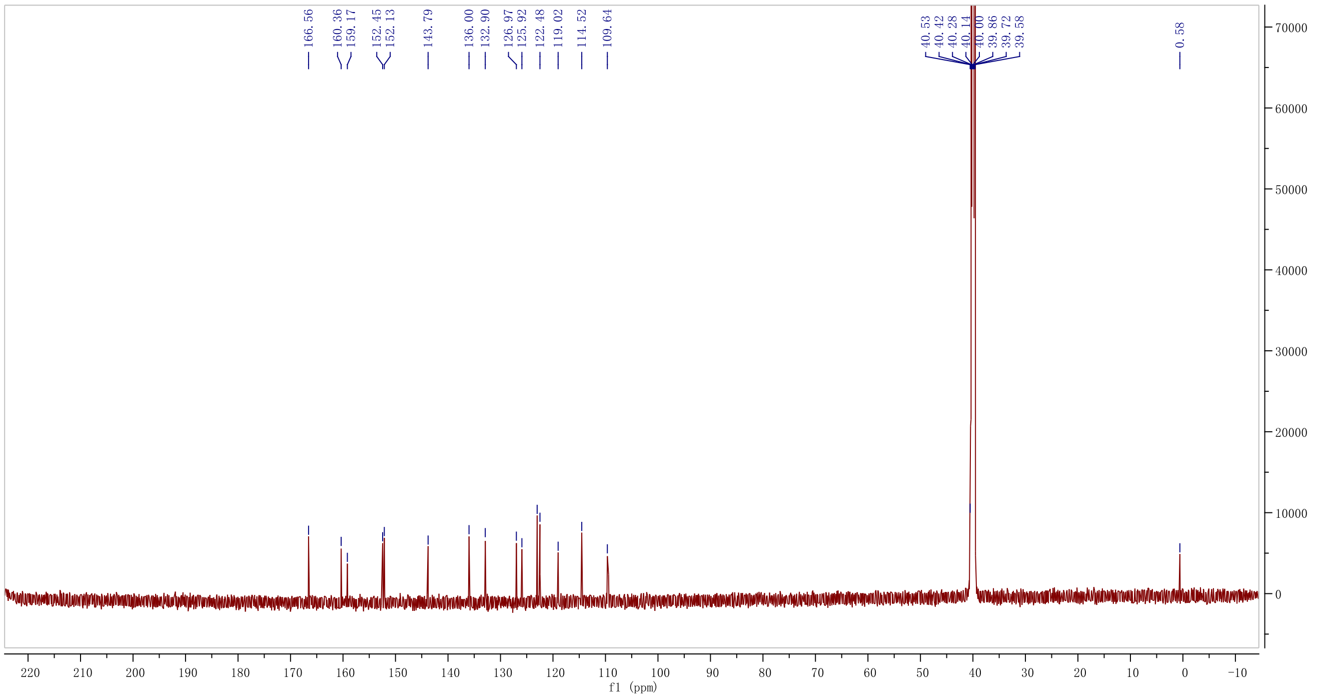

^{1}H NMR and ^{13}C NMR spectra for 3-(benzo[*d*]thiazol-2-yl)-7,8-dihydroxy-2-oxo-2*H*-chromene- 4-carboxamide (22)

^{1}H NMR (600 MHz, DMSO-d_6)

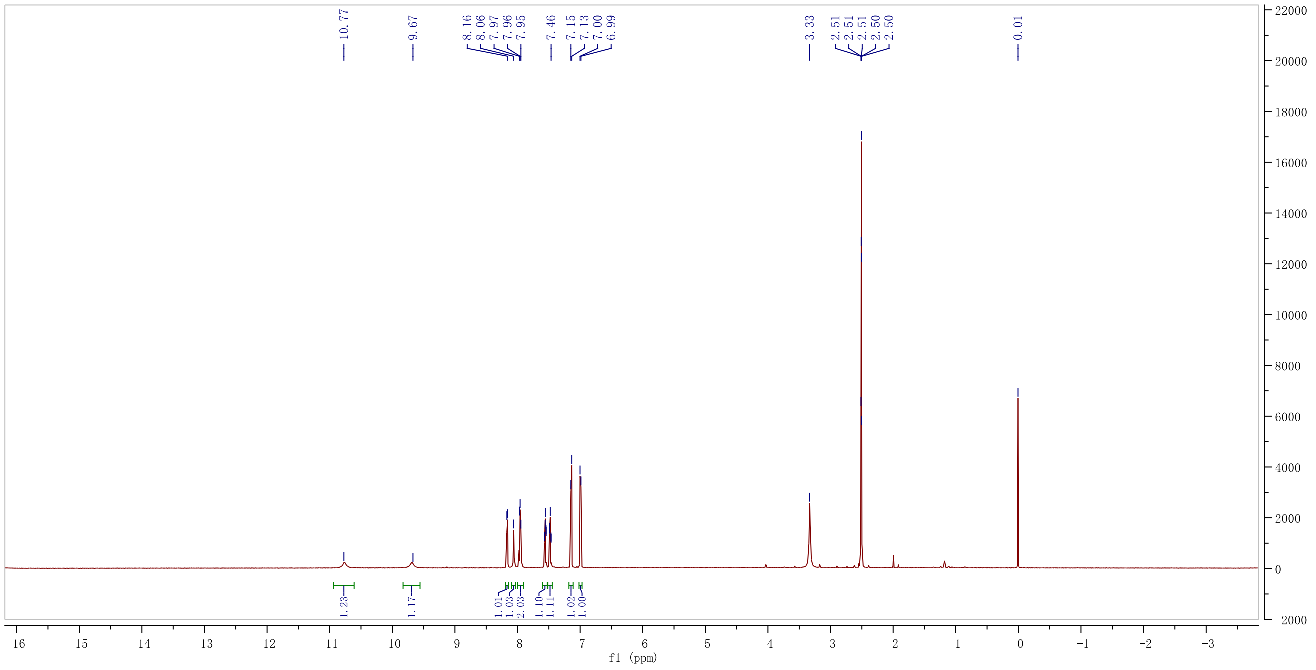

^{13}C NMR (151MHz, DMSO-d_6)

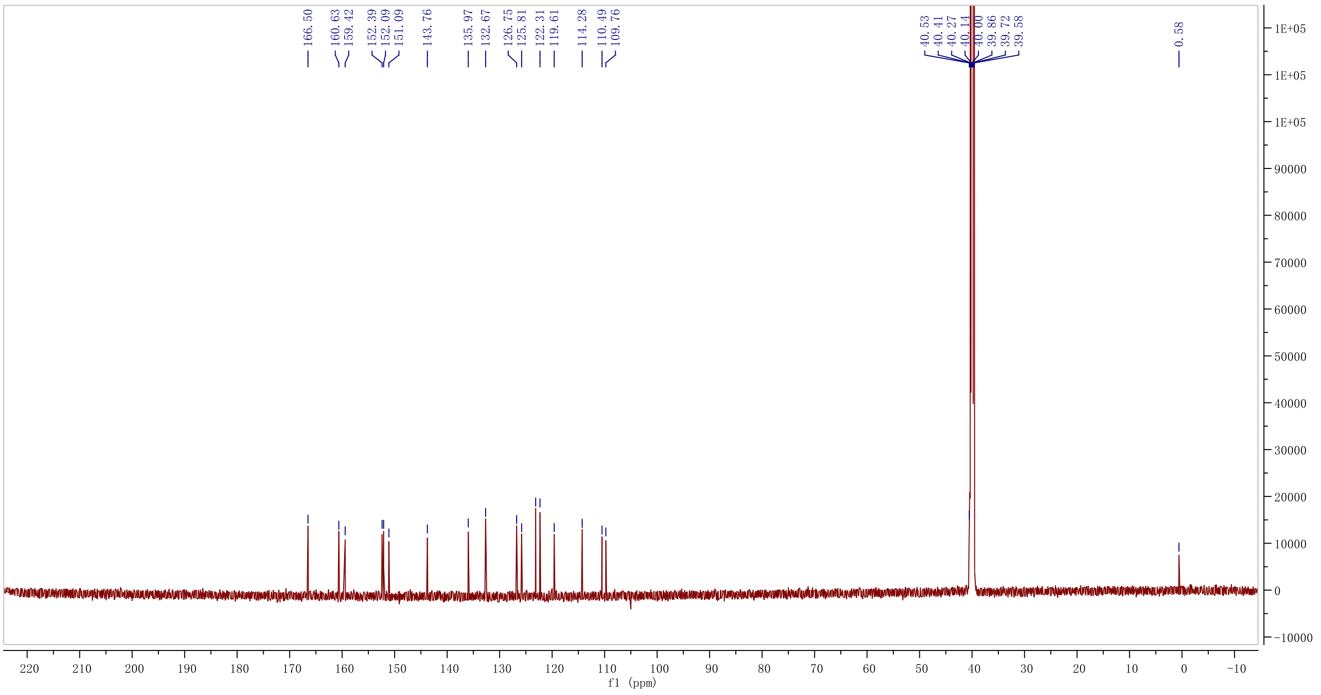

^{1}H NMR and ^{13}C NMR spectra for 3-(benzo[*d*]thiazol-2-yl)-7,8-dihydroxy-4-(1*H*-tetrazol-5-yl)-2*H*-chromen-2-one (23)

^{1}H NMR (600 MHz, DMSO-d_6)

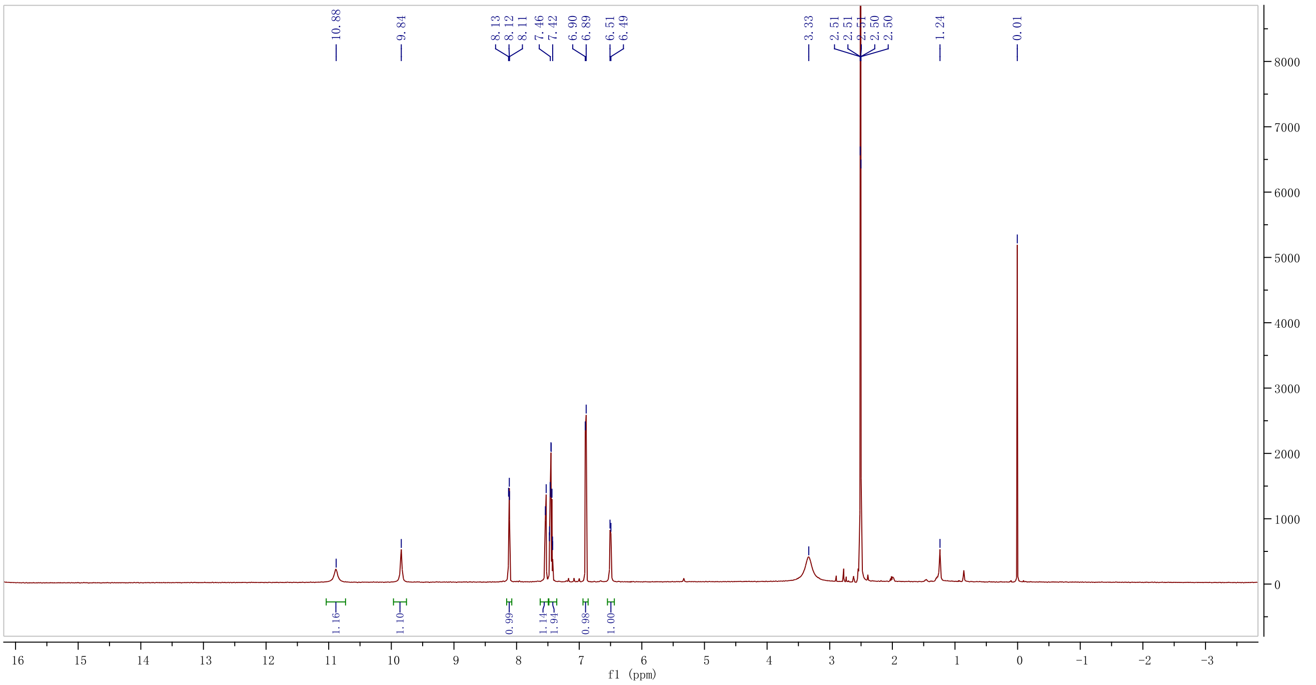

^{13}C NMR (151MHz, DMSO-d_6)

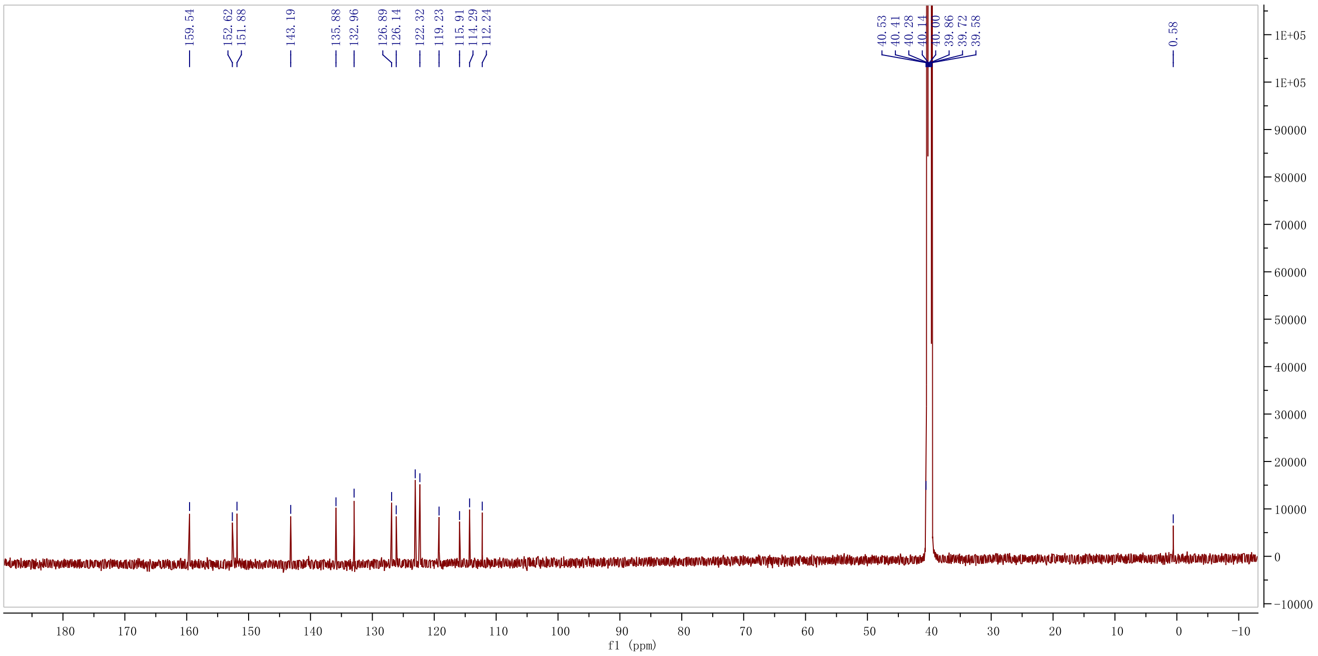

^{1}H NMR and ^{13}C NMR spectra for 7,8-dihydroxy-3-methyl-4-(trifluoromethyl)-2*H*-chromen-2-one (24)

^{1}H NMR (600 MHz, DMSO-d_6)

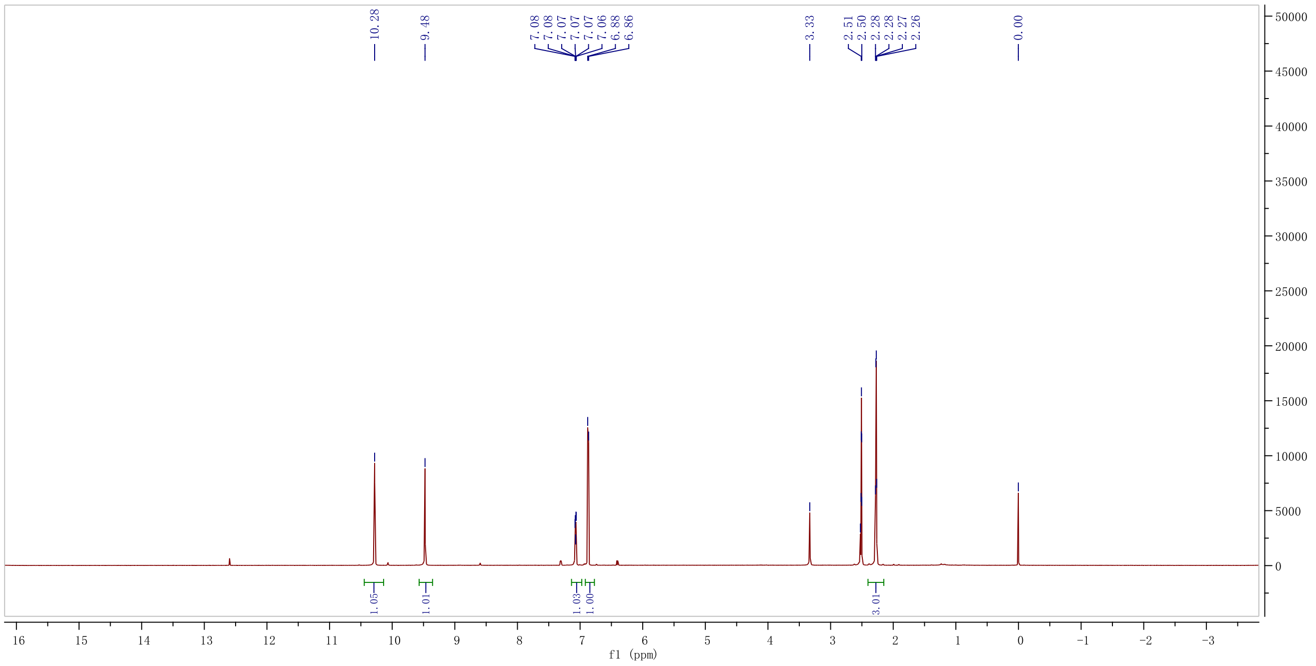

^{13}C NMR (151 MHz, DMSO-d_6)

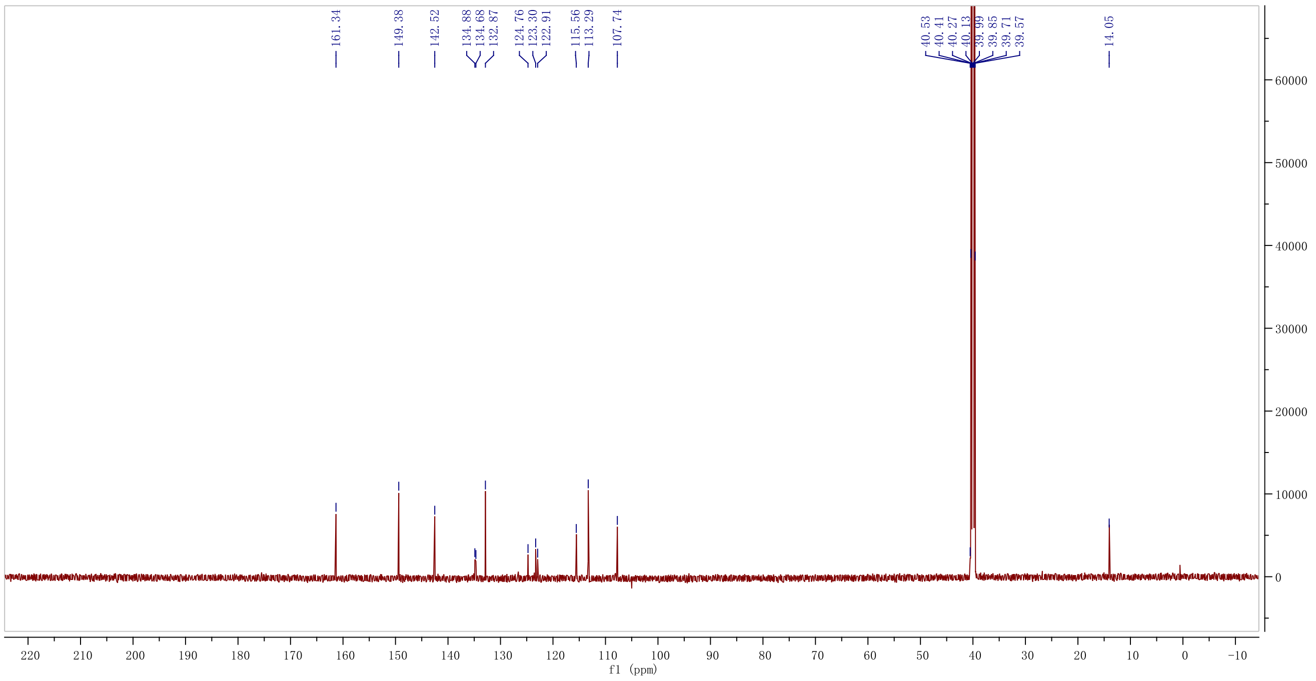

Supplement: Supplementary file 1 [file molecules-23-02476-s001.pdf]
